# The Proteome and Citrullinome of *Hippoglossus hippoglossus* Extracellular Vesicles—Novel Insights into Roles of the Serum Secretome in Immune, Gene Regulatory and Metabolic Pathways

**DOI:** 10.3390/ijms22020875

**Published:** 2021-01-16

**Authors:** Bergljót Magnadóttir, Igor Kraev, Alister W. Dodds, Sigrun Lange

**Affiliations:** 1 Institute for Experimental Pathology at Keldur, University of Iceland, Keldnavegur 3, 112 Reykjavik, Iceland; bergmagn@hi.is; 2 Electron Microscopy Suite, Faculty of Science, Technology, Engineering and Mathematics, Open University, Milton Keynes MK7 6AA, UK; igor.kraev@open.ac.uk; 3 MRC Immunochemistry Unit, Department of Biochemistry, University of Oxford, Oxford OX1 3QU, UK; awdodds@gmail.com; 4 Tissue Architecture and Regeneration Research Group, Department of Biomedical Sciences, University of Westminster, London W1W 6UW, UK

**Keywords:** extracellular vesicles, proteome, citrullinome, peptidylarginine deiminase, deimination/citrullination, complement, pentraxin, immunity, metabolism, gene regulation

## Abstract

Extracellular vesicles (EVs) are lipid bilayer vesicles which are released from cells and play multifaceted roles in cellular communication in health and disease. EVs can be isolated from various body fluids, including serum and plasma, and are usable biomarkers as they can inform health status. Studies on EVs are an emerging research field in teleost fish, with accumulating evidence for important functions in immunity and homeostasis, but remain to be characterised in most fish species, including halibut. Protein deimination is a post-translational modification caused by a conserved family of enzymes, named peptidylarginine deiminases (PADs), and results in changes in protein folding and function via conversion of arginine to citrulline in target proteins. Protein deimination has been recently described in halibut ontogeny and halibut serum. Neither EV profiles, nor total protein or deiminated protein EV cargos have yet been assessed in halibut and are reported in the current study. Halibut serum EVs showed a poly-dispersed population in the size range of 50–600 nm, with modal size of EVs falling at 138 nm, and morphology was further confirmed by transmission electron microscopy. The assessment of EV total protein cargo revealed 124 protein hits and 37 deiminated protein hits, whereof 15 hits were particularly identified in deiminated form only. Protein interaction network analysis showed that deimination hits are involved in a range of gene regulatory, immune, metabolic and developmental processes. The same was found for total EV protein cargo, although a far wider range of pathways was found than for deimination hits only. The expression of complement component C3 and C4, as well as pentraxin-like protein, which were identified by proteomic analysis, was further verified in EVs by western blotting. This showed that C3 is exported in EVs at higher levels than C4 and deiminated C3 was furthermore confirmed to be at high levels in the deimination-enriched EV fractions, while, in comparison, C4 showed very low detection in deimination-enriched EV fractions. Pentraxin was exported in EVs, but not detected in the deimination-enriched fractions. Our findings provide novel insights into EV-mediated communication in halibut serum, via transport of protein cargo, including post-translationally deiminated proteins.

## 1. Introduction

Halibut is a teleost flatfish which belongs to the order Heteresomata (Pleuronectiformes). It is one of the largest teleost fish and endangered due to previous overfishing and slow rate of growth. The Atlantic halibut (*Hippoglossus hippoglossus* L.) is of considerable commercial value for aquaculture, where developmental abnormalities and viability in larval rearing have been one of the major obstacles [1,2]. Furthering understanding of immune, metabolic and developmental processes in commercially viable species, including halibut, is of great importance for the development of biomarkers associated to fish health and improved outcomes in aquaculture.

Peptidylarginine deiminases (PADs) are a calcium-dependent family of enzymes conserved throughout phylogeny with roles in physiological and pathophysiological processes [3,4,5,6]. PADs catalyse protein deimination/citrullination, which is an irreversible post-translational modification of protein arginine to citrulline, leading to structural and functional changes in target proteins [3,6,7]. Deimination can affect protein–protein interactions, as it modifies the protein structure and can cause protein denaturation or affect hydrogen bond formation [5,8]. Deimination can furthermore facilitate protein moonlighting, allowing one protein to carry out various functions within one polypeptide chain [9]. Intrinsically disordered proteins and β-sheets are most prone to undergo deimination and the position of the arginine within the protein plays roles as well [6,8,10]. While in fish, only one PAD form is present [11,12,13,14], mammals contain five tissue-specific PAD isozymes, with varying preferences for target proteins [3,4,5]. In other phyla, such as reptiles and birds, only three PAD forms are described [3,15,16], and PAD homologues are identified lower in the phylogeny tree [17], including in bacteria [18,19], fungi [20], parasites [21], as well as in Crustacea [22], Merostomata [23] and Mollusca [24]. PAD-mediated protein deimination has been reported in a range of taxa throughout the phylogeny tree, both in ontogeny, serum and plasma, as well as forming part of extracellular vesicle (EV) protein cargo [12,13,14,16,22,23,24].

EVs are lipid-bilayer vesicles in the size range of 50–1000 nm, released from most cells and participate in cellular communication in physiology and pathological processes. EVs are classified into small EVs (“exosomes”, <100 nm) and larger EVs (“microvesicles” 100–1000 nm), which are released from cells via different biogenesis pathways, including exocytosis or membrane blebbing [25,26]. Roles for PADs in the modulation of EV release have furthermore been described [27,28,29]. EVs carry a range of cargo, including proteins, enzymes, genetic material, long non-coding RNAs and microRNAs, derived from the cells of origin [25,26,27,28,29,30,31,32,33]. Protein EV cargo can furthermore consist of post-translationally modified proteins, which possibly contribute differently to cellular communication compared with non-modified protein forms. Therefore, it may be of considerable interest to gain insight into differences in such protein cargo in serum-EVs to further understanding of post-translational modifications (PTMs) in cellular communication.

While EV research has been an exponentially expanding field in the past decade in relation to human disease, less is known about EV communication in other taxa. The comparative field of EV research has recently been growing, including by studies from our group [14,16,19,22,23,24,32,33,34,35,36,37,38,39,40]. Therefore, there is currently great interest in expanding EV studies, also in relation to teleost fish and biomarker discovery for aquaculture [32,33,38,41]. Furthermore, fundamental research into EV communication across the phylogeny tree will allow for increased understanding of EV-mediated pathways in evolution.

This study aimed at characterising EVs from halibut sera, assessing both total proteomic cargo and deiminated protein cargo to gain insights into putative roles for protein deimination in the serum secretome.

## 2. Results

### 2.1. EV Profiling from Halibut Sera

**Figure 1:**
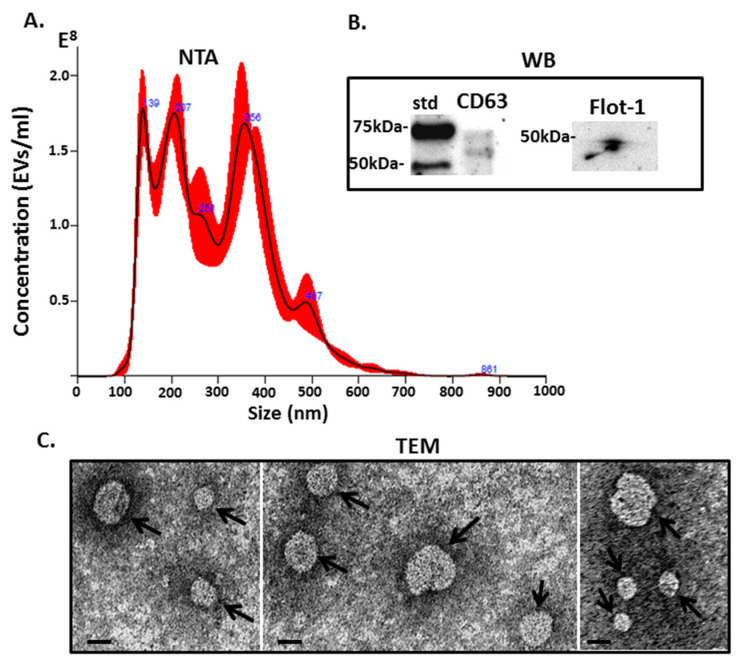
Halibut serum-extracellular vesicles (EV)s were characterised by: (**A**) Nanoparticle tracking analysis (NTA), showing size distribution profiles of EVs in the size range of 50–600 nm, with the modal size of vesicles at 138 nm; (**B**) Western blotting (WB) analysis shows that the EVs are positive for CD63 and Flotillin-1; (**C**) Transmission electron microscopy (TEM) showing EV morphology—see arrows pointing at EVs (scale bar is indicated at 20 nm).

Halibut serum EVs were characterised by NTA, revealing a poly-dispersed EV population in the size range of 50–600 nm, with the modal size of EVs falling at 138 nm (Figure 1A). The EVs were further characterised for two EV specific markers, CD63 and Flotillin-1 and found positive for both (Figure 1B). EV morphology was further confirmed by transmission electron microscopy (TEM), revealing typical EV morphology (see arrows) and confirming a polydispersed population (Figure 1C).

### 2.2. The Proteome and Citrullinome of Halibut Serum EVs

**Figure 2:**
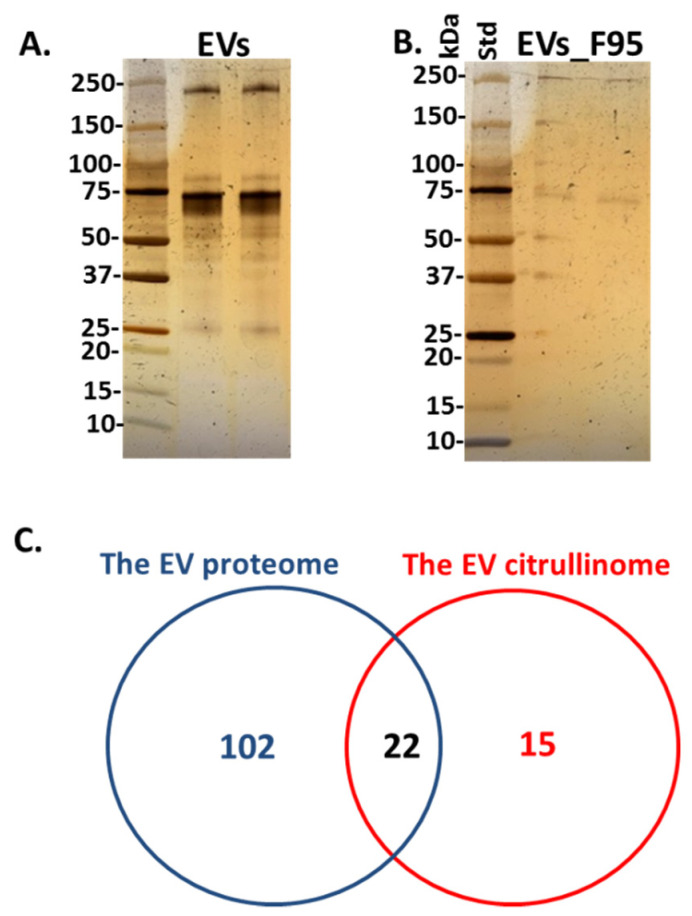
The proteome and citrullinome of halibut serum-EVs. Silver-stained gels for: (**A**) total protein cargo in EVs and (**B**) F95 enriched (deiminated/citrullinated) proteins from EVs. The protein standard (std) is indicated in kilodaltons (kDa). (**C**) Venn diagram shows the number of candidate protein hits identified as cargo in total serum EVs (“The serum EV proteome”) as well as deiminated protein hits in EV cargo (the serum “EV citrullinome”).

Total protein content, as well as F95 enriched protein content, representative of deiminated protein cargo in EVs (the “EV-citrullinome”), was identified by LC-MS/MS analysis. A range of proteins relating to innate and adaptive immunity, as well as gene regulation and cellular function, were identified as deiminated in EV cargo, and are listed in Table 1 (for full details on LC-MS/MS analysis, see Supplementary Table S1). Total EV protein cargo analysis revealed proteins relating to innate and adaptive immunity, nuclear proteins relating to gene regulation, proteins relating to cellular function and metabolism and are listed in Table 2 (for full details on LC-MS/MS analysis, see Supplementary Table S2). Total serum-EV proteins stained by silver staining are shown in Figure 2A, F95 enriched proteins from serum-EVs are shown in Figure 2B and the number of total EV proteins identified, overlapping with deiminated/citrullinated EV proteins identified are presented in the Venn diagram in Figure 2C.

### 2.3. Complement Component C3, C4 and Pentraxin-Like Protein Verified in Halibut EVs and F95 Enriched EV Protein Cargo Fractions Using Western Blotting

**Figure 3:**
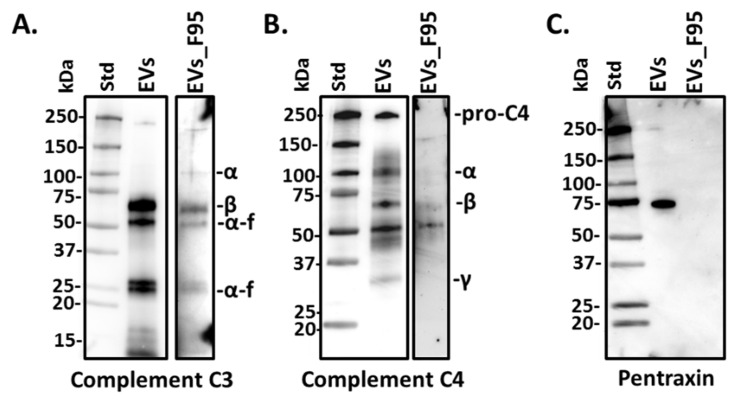
Complement component C3, C4 and pentraxin-like protein in halibut EVs and F95 enriched EV fractions. Western blotting showing (**A**) complement component C3 detection in total protein cargo of halibut serum-EVs (“EVs”) and in F95-enriched protein fractions from serum-EVs (“EVs_F95”), C3 α- and β-chains, as well as α-fragment (α-f) are indicated; (**B**) complement component C4 detection in total protein cargo of serum-EVs (“EVs”) and lower detection observed in F95-enriched EV protein fractions (“EVs_F95”), C4 α-, β- and γ-chains are indicated; (**C**) pentraxin-like protein detection in total EV protein cargo (“EVs”), which was not detected in the F95-enriched EV protein fractions (“EVs_F95”).

Three candidate proteins which were identified as part of EV total protein cargo by LC-MS/MS, namely complement component C3, C4 and pentraxin-like protein, were further assessed by western blotting in halibut serum-EVs (Figure 3A–C). Both total EV protein cargo as well as the F95 enriched protein cargo were assessed, using halibut-specific C3, C4 and pentraxin-like protein antibodies, respectively, which had previously been generated and validated in our laboratories [13,42]. Here, complement component C3 was verified to be present in total EV protein cargo, where it was strongly detected by western blotting, as well as at lower levels in the deiminated (F95-enriched) protein cargo (Figure 3A). This confirmed the hits identified by the LC-MS/MS analysis, showing that C3 is exported in EVs both in normal and deiminated form (Table 1 and Table 2). Complement component C4 was also confirmed to be exported in total EV cargo by western blotting, albeit at lower levels than C3, in accordance with the LC-MS/MS findings which identified C4 as a hit in total EV cargo. C4 was seen only at very low levels in deiminated form in the F95-enriched EV fraction by Wwestern blotting (Figure 3B), and was not identified as part of the F95-enriched cargo by LC-MS/MS. Pentraxin-like protein was strongly detected in total EV protein cargo by western blotting, but not in the F95-enriched EV protein fractions (Figure 3C), in accordance with the results from the LC-MS/MS analysis, which only detected pentraxin in total EV cargo (Table 2).

### 2.4. Protein–Protein Interaction Network Analysis for Halibut Serum-EV Protein Cargo: Deiminated and Total Protein Cargo

#### 2.4.1. Protein Interaction Networks Enriched for Halibut Serum-EV Deiminated/Citrullinated Protein Cargo

**Figure 4:**
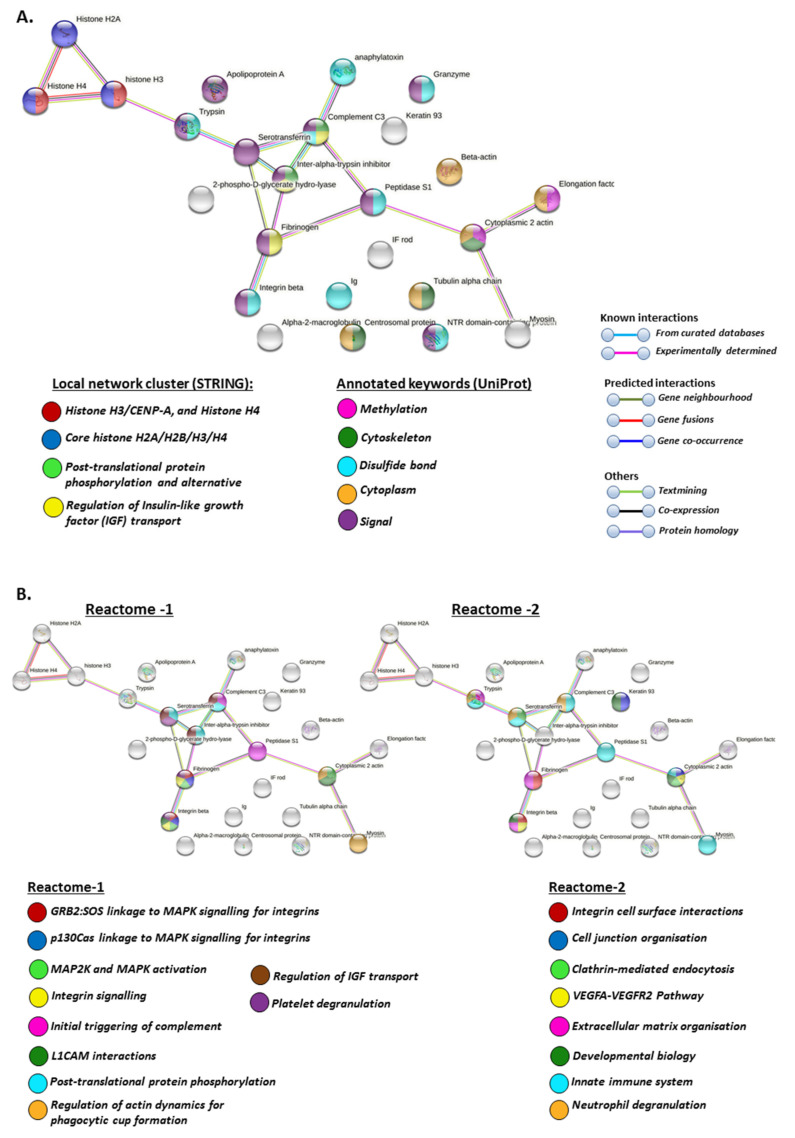

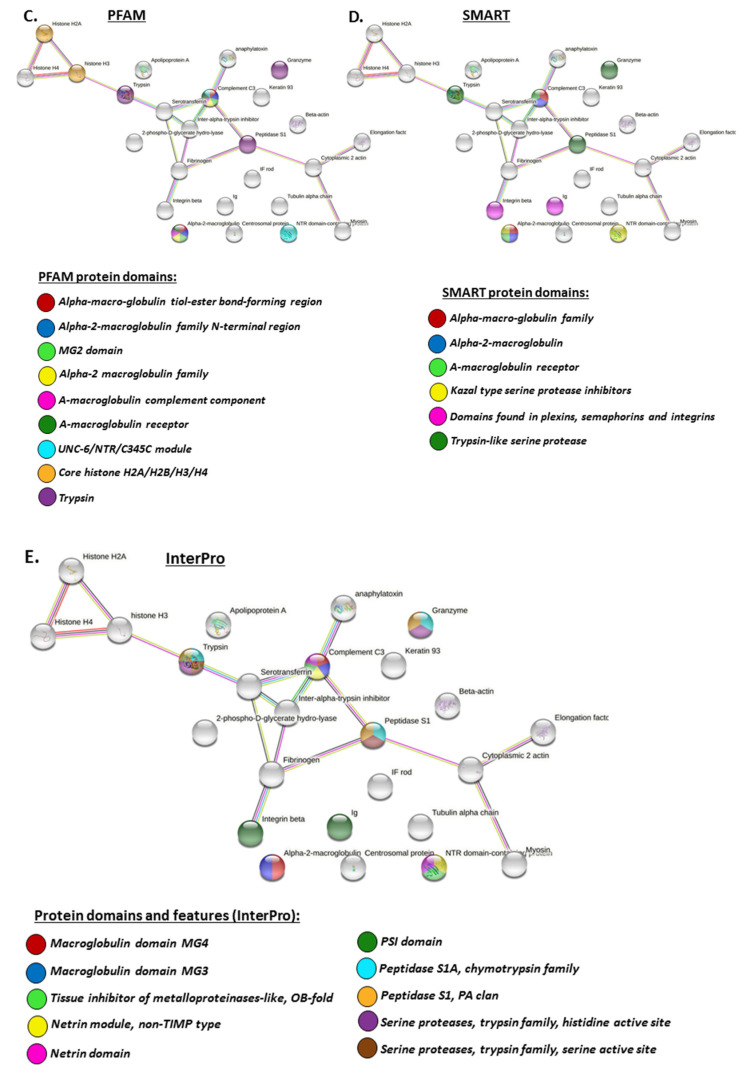
(**A**) Protein interaction networks for deiminated proteins in halibut EVs. Local network clusters and UniProt keywords are indicated by the colour coded nodes. See colour key for nodes and interaction networks in the figure. (**B**) Reactome protein interaction networks for deiminated proteins in halibut EVs. Reactome pathways are indicated by the coloured nodes, as shown in the figure. (**C**,**D**) PFAM and SMART protein interaction networks for deiminated proteins in halibut EVs. PFAM and SMART protein domains are indicated by the coloured nodes, see colour code in the figure. (**E**) InterPro protein interaction networks for deiminated proteins in halibut EVs. InterPro protein domains and features are indicated by the coloured nodes; see colour code in the figure.

For the generation of protein–protein interaction networks to further understanding of putative protein pathways regulated by deimination, deiminated (F95-enriched) protein hits from halibut EVs were assessed by STRING analysis. The protein hits were assessed using the general teleost STRING database, selecting the zebrafish (*Danio rerio*) database as a model database, as no specific database for halibut is available in STRING and zebrafish showed the highest identity with the teleost protein hits identified as deiminated in halibut serum-EVs. The protein–protein interaction networks showed a PPI enrichment *p*-value of 5.15 × 10^−5^, indicating significantly more interactions than expected from a random set of proteins (Figure 4).

Local network clusters enriched in deiminated proteins in EVs included: Histone H3/CENP-A, core histone H2A/H2B/H3/H4 network, post-translational protein phosphorylation and the regulation of IGF transport (Figure 4A). 

UniProt keywords for deiminated proteins identified in serum-EVs included methylation, cytoskeleton, disulphide bond, cytoplasm and signalling (Figure 4A). 

Reactome pathways enriched in deiminated proteins in the serum EVs included GRB2:SOS linkage to MAPK signalling for integrins, p130Cas linkage to MAPK signalling for integrins, MAP2K and MAPK activation, integrin signalling, the initial triggering of complement, L1CAM interactions, post-translational protein phosphorylation, the regulation of actin dynamics for phagocytic cup formation, the regulation of IGF transport, platelet degranulation, integrin cell surface interactions, cell junction organisation, clathrin-mediated endocytosis, VEGFA-VEGFR2 pathway, extracellular matrix organisation, developmental biology, innate immune system and neutrophil degranulation (Figure 4B). 

PFAM protein domains for deiminated proteins identified in the serum EVs included alpha-macro-globulin tiolester bond-forming region, alpha-2-macroglobulin family N-terminal region, MG2 domain, alpha-2 macroglobulin family, a-macroglobulin complement component, a-macroglobulin receptor, UNC-6/NTR/C345C module, core histone H2A/H2B/H3/H4 and trypsin (Figure 4C).

SMART protein domains for deiminated EV proteins included alpha-macroglobulin family, alpha-2-macroglobulin, a-macroglobulin receptor, kazal type serine protease inhibitors, domains found in plexins, semaphorins and integrins and trypsin-like serine protease (Figure 4D).

Protein domains and features (InterPro) for deiminated proteins in serum-EVs included macroglobulin domain MG4 and MG3, tissue inhibitor of metalloproteinases-like, OB-fold, netrin module, non-TIMP type, netrin domain, PSI domain, peptidase S1A, chymotrypsin family, peptidase S1 PA clan, serine proteases trypsin family, histidine active site and serine active site (Figure 4E).

#### 2.4.2. Protein Interaction Networks Enriched for Halibut Serum-EV Total Protein Cargo

**Figure 5:**
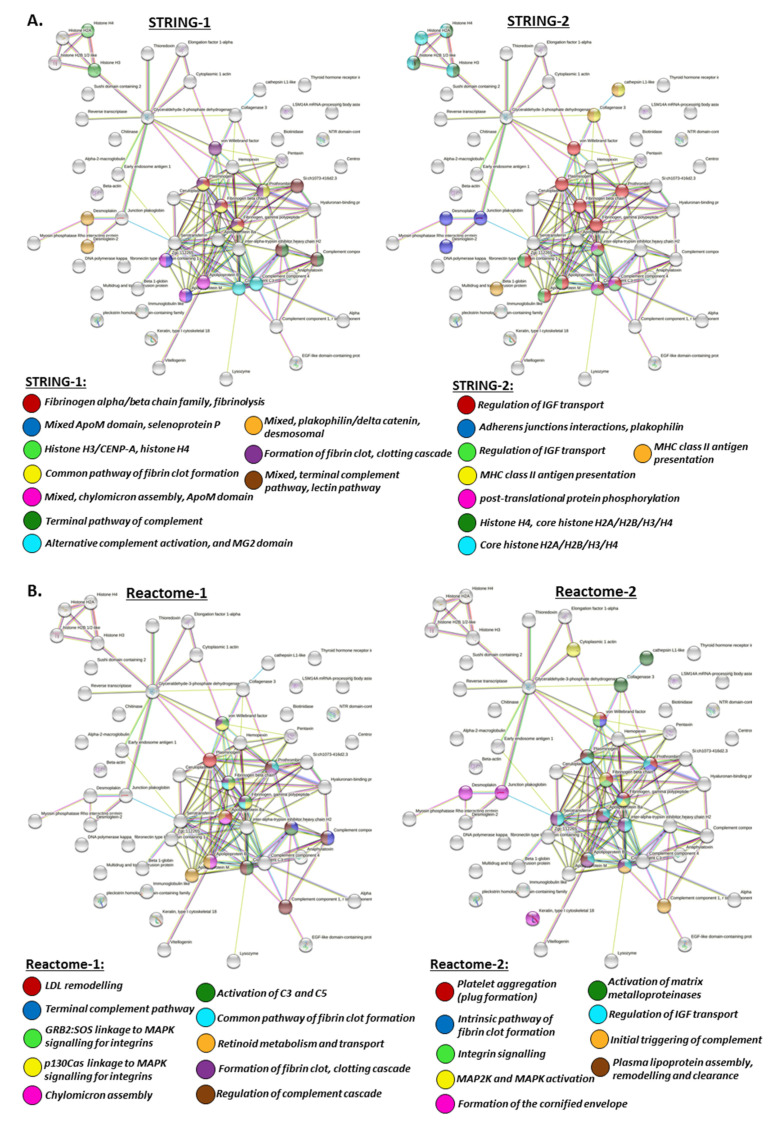

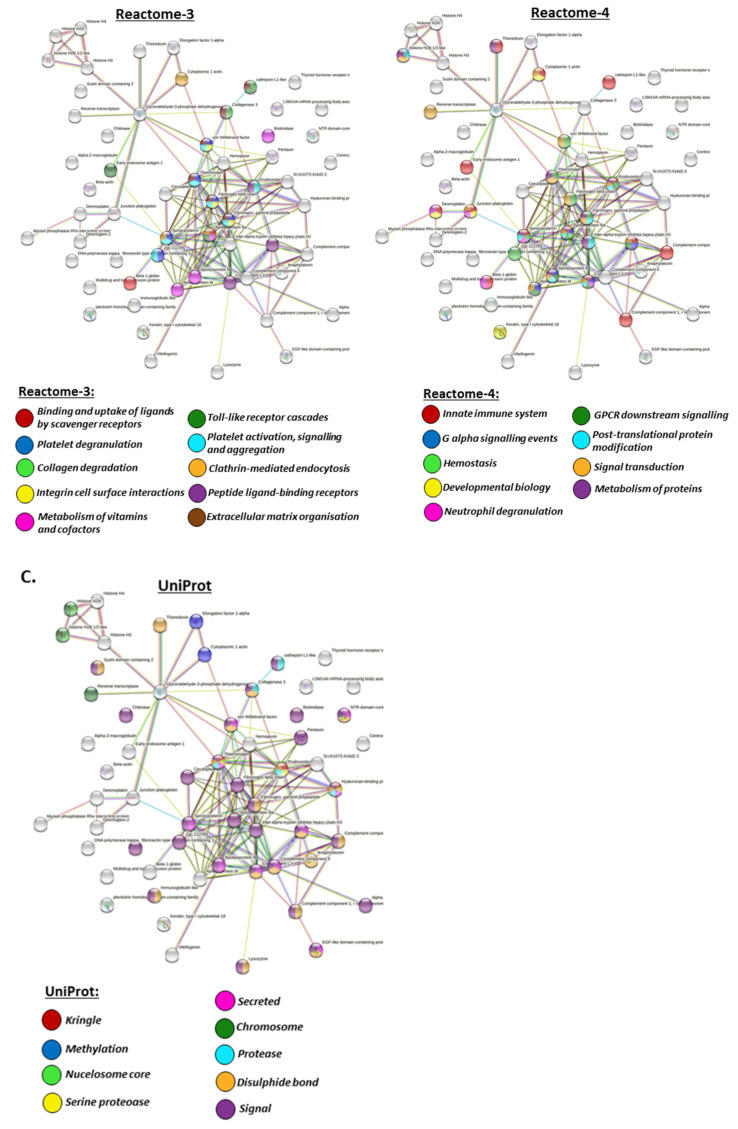

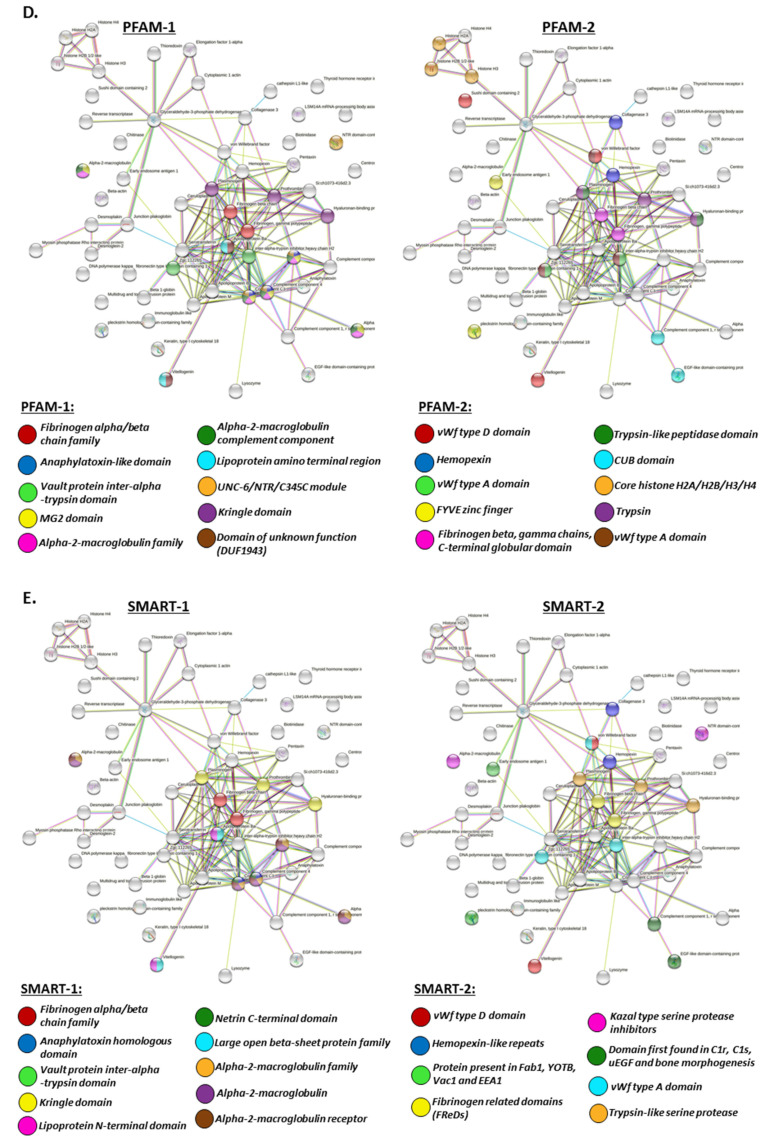

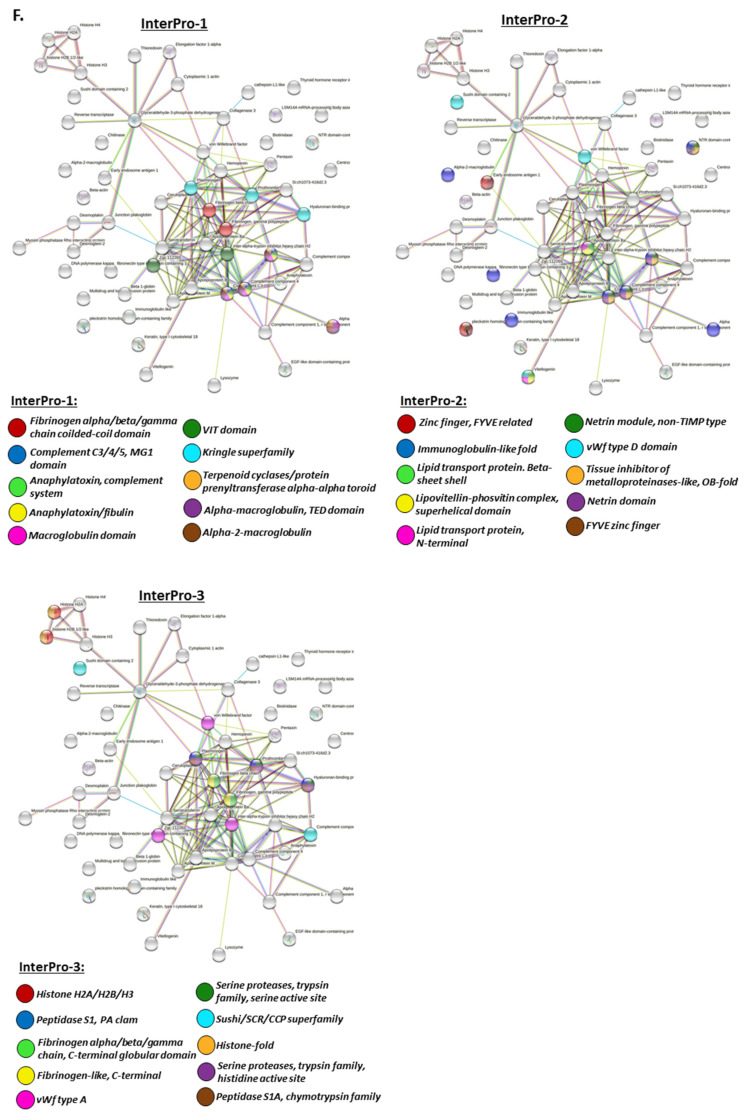
(**A**) Protein interaction networks for total protein cargo in halibut EVs, showing local network clusters. The coloured nodes indicate the different networks, respectively. (**B**) Reactome protein interaction networks for total proteins in halibut EV cargo, showing reactome pathways. Specific reactome pathways are indicated by the coloured nodes, respectively. (**C**) UniProt protein interaction networks for total proteins in halibut EV cargo, showing UniProt keywords. UniProt keywords are indicated by the coloured nodes, respectively. (**D**) PFAM protein interaction networks for total proteins in halibut EV cargo. The specific PFAM protein domains are indicated by the coloured nodes, respectively. (**E**) Protein interaction networks for total proteins in halibut EVs, showing SMART protein domains. The specific SMART protein domains are indicated by the coloured nodes, respectively. (**F**) InterPro protein interaction networks for total proteins in halibut EVs. The specific protein domains and features (InterPro) are indicated by the coloured nodes, respectively.

The same approach for the generation of protein–protein interaction networks, selecting the zebrafish (*D. rerio*) STRING database as a representative database for teleost fish, was also applied for total protein EV cargo identified in halibut, showing a PPI enrichment *p*-value: <1.0 × 10^−16^ for the protein networks generated, indicating significantly more interactions than expected from a random set of proteins (Figure 5).

Local network clusters for total EV protein content included fibrinogen family, fibrinolysis, common pathway of fibrin clot formation, clotting cascade, ApoM domain, selenoprotein P, histone H3/CENP-A, histone H4, terminal pathway of complement, alternative complement activation, MG2 domain, terminal complement pathway, lectin pathway, plakophilin/delta catenin desmosomal, regulation of IGF transport, adherens junctions interactions, MHC class II antigen, MHC class II antigen presentation, post-translational protein phosphorylation, Histone H4, and core histone H2A/H2B/H3/H4 (Figure 5A). 

Reactome pathways for total EV protein cargo included LDL remodelling, plasma lipoprotein assembly, remodelling and clearance, innate immune system, Toll-like receptor cascades, neutrophil degranulation, the regulation of complement cascade, terminal complement pathway, the activation of C3 and C5, the initial triggering of complement, platelet degranulation, platelet activation, GRB2:SOS linkage to MAPK, integrin signalling, integrin cell surface interactions, p130Cas linkage to MAPK signalling for integrins, MAP2K and MAPK activation, the activation of matrix metalloproteinases, chylomicron assembly, the common pathway of fibrin clot formation, the intrinsic pathway of fibrin clot formation, the formation of fibrin clot, clotting cascade, platelet aggregation (plug formation), the formation of the cornified envelope, the regulation of IGF transport, the binding and uptake of ligands by scavenger receptors, collagen degradation, the metabolism of vitamins and cofactors, retinoid metabolism and transport, clathrin-mediated endocytosis, peptide ligand-binding receptors, extracellular matrix organisation, G alpha signalling events, hemostasis, signalling and aggregation, developmental biology, GPCR downstream signalling, post-translational protein modification, signal transduction, metabolism of proteins (Figure 5B). 

UniProt keywords for total EV protein content included methylation, kringle, nucleosome core, serine protease, secreted, chromosome, protease, disulphide bond, signalling (Figure 5C). 

PFAM protein domains for total EV protein cargo included fibrinogen alpha/beta chain family, anaphylatoxin-like domain, vault protein inter-alpha-trypsin domain, MG2 domain, alpha-2-macroglobulin family, alpha-2-macroglobulin complement component, lipoprotein amino terminal region, UNC-6/NTR/C345C module, kringle domain, domain of unknown function (DUF1943), vWf type A domain, vWf type D domain, hemopexin, FYVE zinc finger, fibrinogen beta, gamma chains, C-terminal globular domain, trypsin-like peptidase domain, CUB domain, core histone H2A/H2B/H3/H4, and trypsin (Figure 5D). 

SMART protein domains for total EV protein cargo included fibrinogen alpha/beta chain family, anaphylatoxin homologous domain, vault protein inter-alpha-trypsin domain, kringle domain, lipoprotein N-terminal domain, netrin C-terminal domain, large open beta-sheet protein family, alpha-2-macroglobulin family, alpha-2-macroglobulin receptor, vWf type A domain, vWf type D domain, hemopexin-like repeats, protein present in Fab1, YOTB, Vac1 and EEA1, fibrinogen related domains (FReDs), kazal type serine protease inhibitors, domain first found in C1r, C1s, uEGF and bone morphogenesis, and trypsin-like serine protease (Figure 5E).

Protein domains and features (InterPro) identified for total EV cargo included fibrinogen alpha/beta/gamma chain coiled-coil domain, complement C3/4/5, MG1 domain, anaphylatoxin, anaphylatoxin/fibulin, complement system, macroglobulin domain, alpha-macroglobulin TED domain, alpha-2-macroglobulin, VIT domain, kringle superfamily, terpenoid cyclases/protein prenyltransferase alpha-alpha toroid, zinc finger, FYVE related, FYVE zinc finger, immunoglobulin-like fold, lipid transport protein, beta-sheet shell, lipovitellin-phosvitin complex, superhelical domain, lipid transport protein, netrin domain, netrin module non-TIMP type, vWf type A and vWf type D domain, tissue inhibitor of metalloproteinases-like OB-fold, histone H2A/H2B/H3, histone-fold, peptidase S1, PA clam, fibrinogen alpha/beta/gamma chain C-terminal globular domain, fibrinogen-like C-terminal, serine proteases, trypsin family serine active site and histidine active site, sushi/SCR/CCP superfamily, and peptidase S1A chymotrypsin family (Figure 5F).

## 3. Discussion

This is the first study to assess EV profile signatures in halibut biofluids, identifying both total serum-EV protein cargo as well as deiminated protein cargo in serum-EVs. The size profiling of halibut serum-EVs by NTA showed vesicles in the range of 50–600 nm, which indicates a higher amount of larger EVs compared with human EVs, which typically fall in the size range of 30–300 nm. In comparison, while few teleost fish have been profiled for EVs, cod (*Gadus morhua*), serum-EVs were found to be in the size range of mainly 50–300 nm [33,38], while cod mucus-EVs are in the size range of 50–500 nm [32]. In other taxa across the phylogeny tree, differences in plasma or serum EV size profiles have indeed been reported. In elasmobranches (nurse shark *Ginglymostoma cirratum*) a higher abundance of small EVs in the 10–200 nm size range was observed [14]; in a group of eight pelagic seabird species, some species-specific differences were reported showing plasma-EVs at 50–200 nm size range for some birds and others showing larger EVs at 250–500 nm [37], while in reptile (alligator—*Alligator mississippiesis*), plasma EVs were in the size range of 50–400 nm [16]. In llama (*Lama glama*), plasma-EVs were reported at 40–400 nm [34], while *Bos taurus* plasma-EV showed size profiles of 70–500 nm [35]. Naked mole-rat (*Heterocephelus glaber*) plasma shows similar EV size profiles as human plasma at 50–300 nm [38], as does rat (*Rattus norvegicus*) plasma at 50–250 nm [43]. In sea mammals, such as pinnipeds and cetaceans, serum-EVs were observed at 50–600 nm in seals [40], similar to as observed in halibut in the current study. In four species of whale, EV profiles were seen in the ranges of 50–500 (minke whale *Balaenoptera acutorostrata*), 50–400 (fin whale *Balaenoptera physalus*), 80–300 (humpback whale *Megaptera novaeangliae*) and 90–300 nm (Cuvier’s beaked whale *Ziphius cavirostris*), respectively, while orca serum-EVs (*Orcinus orca*; dolphin family) were reported at 30–500 nm [39]. Reports of EV profiling of haemolymph from species lower in the phylogeny tree include Crustacea (lobster *Homarus americanus*) with EVs in the 10–500 nm size range (with the majority of EVs being small in the 22–115 nm size range) [22]; Mollusca haemolymph EVs at 50–300 nm (blue mussel, *Mytilus edulis*), 30–300 nm (soft shell clam *Mya arenaria*), 90–500 nm (Eastern oyster *Crassostrea virginica*) and 20–300 nm (Atlantic jacknife clam *Ensis leei*), respectively [24]; Arthropoda (horseshoe crab *Limulus polyphemus*) EVs at 20–400 nm (with the majority of EVs falling within 40–123 nm) [23]. In the protozoa *Giardia intestinalis*, two distinct size populations of EVs have been described (20–80 nm and 100–400 nm, respectively), which display different functions in host–pathogen interactions [21]. In Gram-negative and Gram-positive bacteria, with EV profiles described at 10–600 nm and 60–400 nm, respectively, EV profiles were shown to change in response to drug-treatment both with respect to size profile and EV cargo content [19,44]. This does indicate that EV size profiles differ between taxa and this may, amongst others, also have effects on EV cargo content, including proteomic, post-translationally modified proteomic cargo, as well as other genomic and non-coding RNA and mitochondrial-derived cargo [45]. Indeed, in teleost, it has been reported that changes in EV numbers and EV deimination protein and microRNA cargo can be a biomarker for environmental temperature factors [33] and, in response to other stressors, teleost plasma EVs have been found enriched with Hsp70 [46] and selected micro-RNAs [47]. In human parasitic disease, EV profiles can also be indicative of infection status [48]. Therefore, the characterisation of EVs across a wide range of taxa further highlights their potential for biomarker application or “EV-fingerprinting” for the assessment of animal health. 

Analysing both whole proteomic and the deiminated protein content of halibut serum-EVs in the current study, some differences were found in protein-interaction pathways, while overall both the whole proteome and the EV-citrullinome involved a number of immune, metabolic and gene regulatory pathways. 

When assessing protein-protein interaction networks for EVs enriched in deiminated proteins, these related to local network clusters for deiminated proteins in serum-EVs included histone H3/CENP-A, core histone H2A/H2B/H3/H4 network, post-translational protein phosphorylation and the regulation of IGF transport. In relation to such networks, UniProt keywords for deiminated proteins identified in serum-EVs included methylation, cytoskeleton, disulphide bond, cytoplasm and signalling. Reactome pathways enriched in deiminated proteins in the serum EVs included GRB2:SOS linkage to MAPK signalling for integrins, p130Cas linkage to MAPK signalling for integrins, MAP2K and MAPK activation, integrin signalling, initial triggering of complement, L1CAM interactions, post-translational protein phosphorylation, regulation of actin dynamics for phagocytic cup formation, regulation of IGF transport, platelet degranulation, integrin cell surface interactions, cell junction organisation, clathrin-mediated endocytosis, VEGFA–VEGFR2 pathway, extracellular matrix organisation, developmental biology, innate immune system and neutrophil degranulation. Correspondingly, PFAM protein domains for deiminated proteins identified in the serum EVs included alpha-macro-globulin tiolester bond-forming region, alpha-2-macroglobulin family N-terminal region, MG2 domain, alpha-2 macroglobulin family, alpha-macroglobulin complement component, alpha-macroglobulin receptor, UNC-6/NTR/C345C module, core histone H2A/H2B/H3/H4 and trypsin. SMART protein domains for deiminated EV proteins included alpha-macroglobulin family, alpha-2-macroglobulin, alpha-macroglobulin receptor, kazal-type serine protease inhibitors, domains found in plexins, semaphorins and integrins and trypsin-like serine. Protein domains and features (InterPro) for deiminated proteins in serum-EVs included macroglobulin domain MG4 and MG3, the tissue inhibitor of metalloproteinases-like OB-fold, netrin module, non-TIMP type, netrin domain, PSI domain, peptidase S1A, chymotrypsin family, peptidase S1 PA clan, serine proteases trypsin family, histidine active site and serine active site.

In comparison with deiminated EV protein content, more pathways were revealed for serum-EV total protein content, as would be expected due to only some of the proteins in the EV cargo being candidates for post-translational deimination and exported in EVs in deiminated form. Assessing protein interaction networks for total protein EV content showed local network clusters for fibrinogen family, fibrinolysis, the common pathway of fibrin clot formation, clotting cascade, ApoM domain, selenoprotein P, histone H3/CENP-A, histone H4, the terminal pathway of complement, alternative complement activation, MG2 domain, terminal complement pathway, lectin pathway, plakophilin/delta catenin desmosomal, the regulation of IGF transport, adherens junctions interactions, MHC class II antigen, MHC class II antigen presentation, post-translational protein phosphorylation, Histone H4, and core histone H2A/H2B/H3/H4.

The reactome pathways for total EV protein cargo included LDL remodelling, plasma lipoprotein assembly, remodelling and clearance, innate immune system, Toll-like receptor cascades, neutrophil degranulation, the regulation of the complement cascade, the terminal complement pathway, the activation of C3 and C5, the initial triggering of complement, platelet degranulation, platelet activation, GRB2:SOS linkage to MAPK, integrin signalling, integrin cell surface interactions, p130Cas linkage to MAPK signalling for integrins, MAP2K and MAPK activation, the activation of matrix metalloproteinases, chylomicron assembly, the common pathway of fibrin clot formation, the intrinsic pathway of fibrin clot formation, the formation of fibrin clot, clotting cascade, platelet aggregation (plug formation), the formation of the cornified envelope, the regulation of IGF transport, the binding and uptake of ligands by scavenger receptors, collagen degradation, the metabolism of vitamins and cofactors, retinoid metabolism and transport, clathrin-mediated endocytosis, peptide ligand-binding receptors, extracellular matrix organisation, G alpha signalling events, hemostasis, signalling and aggregation, developmental biology, GPCR downstream signalling, post-translational protein modification, signal transduction, and the metabolism of proteins. 

UniProt keywords for total EV protein content included methylation, kringle, nucleosome core, serine protease, secreted, chromosome, protease, disulphide bond, signalling.

PFAM protein domains for total EV protein cargo included fibrinogen alpha/beta chain family, anaphylatoxin-like domain, vault protein inter-alpha-trypsin domain, MG2 domain, alpha-2-macroglobulin family, alpha-2-macroglobulin complement component, lipoprotein amino terminal region, UNC-6/NTR/C345C module, kringle domain, the domain of unknown function (DUF1943), vWf type A domain, vWf type D domain, hemopexin, FYVE zinc finger, fibrinogen beta, gamma chains, C-terminal globular domain, trypsin-like peptidase domain, CUB domain, core histone H2A/H2B/H3/H4, and trypsin.

SMART protein domains for total EV protein cargo included fibrinogen alpha/beta chain family, anaphylatoxin homologous domain, vault protein inter-alpha-trypsin domain, kringle domain, lipoprotein N-terminal domain, netrin C-terminal domain, large open beta-sheet protein family, alpha-2-macroglobulin family, alpha-2-macroglobulin receptor, vWf type A domain, vWf type D domain, hemopexin-like repeats, protein present in Fab1, YOTB, Vac1 and EEA1, fibrinogen related domains (FReDs), kazal type serine protease inhibitors, domain first found in C1r, C1s, uEGF and bone morphogenesis, and trypsin-like serine protease.

Protein domains and features (InterPro) identified for total EV protein cargo included fibrinogen alpha/beta/gamma chain coiled-coil domain, complement C3/4/5, MG1 domain, anaphylatoxin, anaphylatoxin/fibulin, complement system, macroglobulin domain, alpha-macroglobulin TED domain, alpha-2-macroglobulin, VIT domain, kringle superfamily, terpenoid cyclases/protein prenyltransferase alpha-alpha toroid, zinc finger, FYVE related, FYVE zinc finger, immunoglobulin-like fold, lipid transport protein, beta-sheet shell, lipovitellin-phosvitin complex, superhelical domain, lipid transport protein, netrin domain, netrin module non-TIMP type, vWf type A and vWf type D domain, the tissue inhibitor of metalloproteinases-like OB-fold, histone H2A/H2B/H3, histone-fold, peptidase S1, PA clam, fibrinogen alpha/beta/gamma chain C-terminal globular domain, fibrinogen-like C-terminal, serine proteases, trypsin family serine active site and histidine active site, sushi/SCR/CCP superfamily, peptidase S1A chymotrypsin family.

Proteomic analysis using LC-MS/MS, identified a range of innate and adaptive immune proteins to be exported in serum-EVs, including in deiminated form, as listed above. This also included a range of complement components, whereof C3 and C5 were detected as deiminated in serum EVs, while in total EV cargo, C1, C3, C4, C5, C6, C7, C8 and C9 were also identified as hits, as well as factor B and factor H. This correlates with previous findings reporting C3 to be deiminated in teleost fish, both in halibut and cod [13,32,33]. Furthermore, a proteomic analysis of deiminated target proteins in halibut serum identified C5, C7, C8 C9 and C1-inhibitor to be deiminated in whole halibut serum [13]. These findings, and the current study, indicate that not all complement components are exported in EVs in deiminated form, and some are found in deiminated form only in whole serum, while being exported in non-deiminated form in serum-EVs. Recent studies assessing protein deimination across the phylogeny tree have indeed identified various complement components as deimination candidates in a range of taxa [14,16,32,33,34,35,37,39,40]. Furthermore, C5 has been verified to be a deimination candidate by bacterial arginine deiminase, allowing for immune modulation of the host and bacterial immune evasion [18].

In the current study we furthermore evaluated by western blotting some key complement proteins identified by LC/MS-MS in EV total protein cargo and deimination-enriched protein cargo. For this purpose, we used halibut-specific antibodies against C3, C4 and pentraxin-like protein, previously developed and described by our group [13,42]. Using western blotting analysis, we verified the presence of C3, C4 and pentraxin-like protein in halibut serum-EVs, showing that these are indeed exported in EVs, as also identified by LC-MS/MS anlaysis. The C3 antibody also reacted strongly with the F95 enriched protein eluate from the serum EVs, while a lower signal was seen for C4, indicating that C3 is present at higher levels in deiminated form in serum-EVs, compared with C4. Pentraxin-like protein was only observed in total protein cargo of serum-EVs, but not the F95 enriched serum-EV eluate and this corresponds with the LC-MS/MS analysis which revealed hits with a pentaxin for the total protein cargo analysis of serum-EVs, but not the F95-enriched fraction. Our current findings in halibut serum-EV cargo also correspond to our previous analysis on serum EVs and mucus EVs in Atlantic cod, where C3 was detected at higher levels in serum-EVs than C4, both for total protein as well as in the F95-enriched eluate for a putative deiminated form [32,33,38]. Furthermore, cod serum and mucus EVs were also found to contain pentraxin-like protein (CRP-like), which was not detected in deiminated form in the cod EVs, similar to as observed for pentraxin-like protein in halibut serum-EVs in the current study.

Overall, our and others’ findings indicate that the complement system can be modulated by deimination both by the host and by pathogen interactions. Understanding of post-translational regulation of complement components via deimination is still in its infancy and requires in depth investigation as deimination may facilitate multifaceted functions of complement proteins in immunity and tissue remodelling in health and disease, also across phylogeny. Such regulation via deimination may furthermore allow for targeted modulation in relation to a range of pathological processes, including infection and autoimmune diseases, where PADs, EVs and the complement system all play important roles.

Besides differences in EV cargo for complement components, proteins that were only identified in whole protein cargo (and not in the F95 eluate) related to a range of innate and adaptive immune factors as well as metabolic and gene regulatory function. These included Apolipoprotein Bb, Apolipoprotein M, Ig-like domain-containing protein, Ig heavy chain Mem5-like, IGv domain-containing protein, Immunoglobulin light chain, nattectin, SERPIN domain-containing protein, Lysozyme, ceruloplasmin, vitellogenin, apoptosis-stimulating of p53 protein 2 Bcl2-binding protein, plasminogen, keratin, type I cytoskeletal 13-like, EGF-like domain-containing protein, hephaestin-like protein 1, desmoglein-2, carboxypeptidase Q, beta 1-globin, antithrombin-III, collagen alpha-1(XII) chain, desmoplakin, biotinidase, collagenase 3, cathepsin L1-like, prothrombin, putative insulin-like growth factor binding protein, sushi domain-containing protein 2 isoform 2, 14_3_3 domain-containing protein, catechol O-methyltransferase domain-containing protein 1, pleckstrin, hyaluronan-binding protein 2, retrotransposon-derived, FH2 domain-containing protein 1-like, protein-tyrosine-phosphatase, thyroid hormone receptor interactor 11, roundabout-like axon guidance receptor protein 2, protein Z-dependent protease inhibitor-like, myosin phosphatase Rho interacting protein, multidrug and toxin extrusion protein, FH2 domain containing 4, nesprin-2, histone H3-trimethyl-L-lysine(9) demethylase, centrosomal protein of 290 kDa and titin-like protein. These all relate to the protein-interaction networks identified for whole EV protein cargo, listed above and shown in Figure 5.

Furthermore, some protein candidates were only detected in the F95 eluate, indicating that they were exported in deiminated form only in the serum EVs. This included cytoplasmic 2 actin, tubulin alpha chain, keratin 93, trypsin-3-like, centrosomal protein of 162 kDa, 2-phospho-D-glycerate hydro-lyase, integrin beta and myosin_tail_1 domain-containing protein. Compared with a previous analysis from our group on deiminated proteins in whole halibut serum [13], deiminated candidates found here to be exported specifically in EVs are cytoplasmic 2 actin, tubulin alpha chain, centrosomal protein of 162 kDa, 2-phospho-D-glycerate hydro-lyase, integrin beta and myosin_tail_1 domain-containing protein. This indicates that there are differences in deiminated protein cargo in serum-EVs compared with whole serum, and this corresponds to findings from other comparative studies analysing differences in KEGG (Kyoto encyclopedia of genes and genomes) and GO (gene ontology) enrichment pathways for deiminated proteins in whole serum/plasma versus EVs in diverse taxa, including in cow, camelid, alligator, rat, naked mole-rat, shark and cod [14,16,33,34,35,36,43]. Furthermore, differences in deimination signatures in whole serum versus EV cargo have been reported to relate to immune/growth trade off in response to environmental temperature in teleost cod [33]. Such findings, including the findings reported in our current study, emphasise that variations in EV cargo, including via the transport of deiminated proteins, may play hitherto under-recognized and important roles in cellular communication in health and disease across the phylogeny tree. 

## 4. Materials and Methods

### 4.1. Fish and Sampling

Blood was collected from four adult halibut (*Hippoglossus hippoglossus* L.; weight 4.5–5.0 kg), which were obtained from the experimental fish farm Fiskeldi Eyjafjardar hf, Thorlakshofn, Iceland (under licence from the Institute for Experimental Pathology, University of Iceland, number #0002 kt-650269—4549, approved by the central animal ethics committee in Iceland (Icelandic Food Regulation Authority, MAST Matvælastofnun). Following 1–3 mL blood collection from a gill vessel, the blot was left to clot overnight at 4 °C, and thereafter serum collection was performed by centrifugation at 750 *g* for 10 min. Serum aliquots of 200 µL were stored at −20 °C until used. The health status of the fish at the fish farm was routinely examined at regular 3 monthly intervals by the Fish Disease Laboratory, Institute for Experimental Pathology, Keldur, Iceland, declaring the fish healthy and disease free.

### 4.2. EV Isolation and Nanoparticle Tracking (NTA) Analysis

EVs were isolated from halibut serum of four individual fish by step-wise centrifugation, according to previously established methods in our laboratory [14,22,39] and according to the guidelines of the International Society for Extracellular Vesicles (ISEV) [49]. Total EV isolates were prepared from the individual 100 μL serum aliquots (*n* = 4), which were diluted 1:5 in Dulbecco’s PBS (DPBS, which had previously been ultrafiltered using a 0.22 μm filter, before use) and then centrifuged at 4000 *g* for 30 min at 4 °C, to ensure the removal of aggregates and apoptotic bodies. The supernatants containing the EVs were collected and ultracentrifuged at 100,000 *g* for 1 h at 4 °C. The EV-enriched pellets were then resuspended in 1 mL DPBS (“washing step”) and ultracentrifuged again at 100,000 *g* for 1 h at 4 °C. The final EV-enriched pellets were then resuspended in 100 µL DPBS and analysed by NTA for size distribution profiles, using the NanoSight NS300 system (Malvern Panalytical Ltd, Malvern, UK), recording five 60 s videos for each sample. The number of particles per frame was kept in-between 40 to 60 and replicate histograms were generated from the videos, using the NanoSight software 3.0 (Malvern), representing mean and confidence intervals of the five recordings for each sample.

### 4.3. Transmission Electron Microscopy (TEM)

EVs were further characterised by Transmission Electron Microscopy (TEM) as follows: A pool of EVs, isolated from serum of the four individual animals as described above, was used for morphological analysis using TEM according to previously described methods [16,23,34]. In brief, 100 mM sodium cacodylate buffer (pH 7.4) was used to resuspend the EVs, which were then placed onto a glow discharged carbon support film on a grid and fixed at room temperature for 1 min in 2.5% glutaraldehyde in 100 mM sodium cacodylate buffer (pH 7.0). For the staining of EVs, 2% aqueous Uranyl Acetate (Sigma, Gillingham, UK) was used for 1 min, thereafter removing the excess stain. EV imaging was performed using a JEOL JEM 1400 transmission electron microscope (JEOL, Tokyo, Japan) operated at 80 kV at a magnification of 30,000× to 60,000×. Digital images were recorded using an AMT XR60 CCD camera (Deben, Bury St. Edmunds, UK).

### 4.4. Proteomic Analysis and Protein Identification

The isolation of deiminated/citrullinated proteins from serum-EVs was carried out by immunoprecipitation, using the Catch and Release^®^ v2.0 Reversible Immunoprecipitation System (Merck, Feltham, UK) according to the manufacturer’s instructions in conjunction with the pan-deimination F95 antibody (MABN328, Merck), which specifically detects proteins modified by citrullination [50]. F95 enrichment was performed overnight at 4 °C on a rotating platform from a pool of sera (*n* = 4 individuals), followed by the elution of the F95 bound proteins under reducing conditions, according to the manufacturer’s instructions (Merck). The F95 eluate was diluted in 2 × Laemmli sample buffer for subsequent SDS-PAGE and western blotting analysis. The total F95 bound protein eluate, as well as total protein from serum-EVs, were also analysed by liquid chromatography–mass spectrometry (LC-MS/MS) (performed by Cambridge Centre for Proteomics, Cambridge, UK), by in-gel digestion, as previously described [32,34]. For the identification of deiminated protein hits, the files were submitted to the Mascot search algorithm (Matrix Science, London, UK) and searched against the UniProt database for Teleostei (CCP_Teleostei Teleostei_20201009; 4085639 sequences; 2121030378 residues). A search was also conducted against a common contaminant database (cRAP 20190401; 125 sequences; 41,129 residues). A significance threshold value of *p* < 0.05 and a peptide cut-off score of 53 were also applied (carried out by Cambridge Proteomics, Cambridge, UK).

In addition to the LC-MS/MS analysis, both total EV proteins and F95 enriched EV proteins were assessed specifically for halibut C3, C4 and pentraxin-like protein content, using mono-specific antibodies, which were previously prepared against these proteins by our group [13,42] (see Section 4.5).

### 4.5. Western Blotting

Serum EVs were pooled (*n* = 4), reconstituted 1:1 in 2 × Laemmli sample buffer and boiled at 100 °C for 5 min before separation by SDS-PAGE, using 4–20% TGX gels (BioRad, Watford, UK). Proteins were blotted onto 0.45 μm nitrocellulose membranes (BioRad, UK) using semi-dry transfer for 1 h at 15 V and even protein transfer was assessed using Ponceau S (Sigma, Gillingham, UK) staining. Membranes were blocked for 1 h at RT in 5% bovine serum albumin (BSA, Sigma) in Tris-buffered saline containing Tween20 (TBS-T). Primary antibody incubation was performed overnight at 4 °C on a shaking platform, diluting the antibodies in TBS-T. EVs were assessed for the EV-specific markers Flotillin-1 (ab41927, 1/1000) and CD63 (ab216130, 1/1000). EV cargo was assessed for halibut pentraxin-like protein (1/1000; [13]), halibut C3 (1/1000; [42]) and halibut C4 (1/1000; [13]), using halibut-specific antibodies previously generated in our laboratory [13,42]. Following primary antibody incubation, the membranes were washed three times in TBS-T and then incubated at room temperature for 1h in either the corresponding anti-mouse IgG (for anti-pentraxin, anti-C3 and anti-C4 antibodies) or anti-rabbit IgG (for CD63 and Flot-1 antibodies) HRP-conjugated secondary antibodies (1/3000; BioRad). Thereafter, the membranes were washed in TBS-T five times for 10 min and then visualised using a UVP BioDoc-IT^TM^ System (Cambridge, UK) in conjunction with ECL (Amersham, Merck, UK).

### 4.6. Silver Staining

Total proteins isolated from serum EVs and the F95-enriched protein eluates from halibut serum EVs were assessed by silver staining following SDS-PAGE in 4–20% gradient TGX gels (BioRad) under reducing conditions. The BioRad Silver Stain Plus Kit (1610449, BioRad) was used to visualise the protein bands according to the manufacturer’s instructions (BioRad).

### 4.7. Protein–Protein Interaction Network Analysis

For the construction of protein–protein interaction networks for deiminated proteins identified in halibut serum-EVs and for total protein content from serum EVs, respectively, STRING analysis (Search Tool for the Retrieval of Interacting Genes/Proteins; https://string-db.org/) was applied. The protein networks were built based on the protein names, using the teleost fish STRING database and using the function of “search multiple proteins”. Settings were as “basic” and “medium confidence”. Colour lines connecting the nodes represent the following evidence-based interactions for the network edges: “known interactions” (this is based on experimentally determined data or curated databases); “predicted interactions” (this is based on gene co-occurrence, gene neighbourhood or gene fusion); “others” (this is based on co-expression, text mining or protein homology (see colour key for lines in Figure 4A). Networks were assessed for local network clusters, reactome pathways, PFAM and SMART protein domains and UniProt keywords. The zebrafish (*Danio rerio*) STRING database was used as representative for Teleostei for the creation of the networks as no specific halibut STRING database is available (due to lack of annotation available for halibut), and *D. rerio* showed the most hit number identity with the proteins identified in halibut EVs.

### 4.8. Statistical Analysis

The Nanosight 3.0 software (Malvern) was used for the generation of NTA curves, which represent mean and standard error of mean (SEM), indicated by confidence intervals. Significance for protein network analysis generated in STRING (https://string-db.org/) was considered as *p* ≤ 0.05.

## 5. Conclusions

This study is the first report of EV profile signatures in halibut, analysing total protein and specifically also deiminated protein cargo in serum-EVs. Halibut serum EVs showed a poly-dispersed population with EVs in the size range of 50–600 nm, positive for phylogenetically conserved EV markers. Proteomic analysis of EV total protein cargo revealed 124 protein hits and 37 deiminated protein hits, whereof 15 hits were particularly identified in deiminated form only. Protein interaction network analysis revealed GO pathways for EV mediated protein cargo transport, relating to a range of gene regulatory, immune, metabolic and developmental processes, some of which were enriched for deiminated proteins. Further assessment of key immune related proteins—complement components C3, C4 and pentraxin—identified that C3 is exported in serum-EVs at higher levels than C4, also in deiminated form, while pentraxin was found in whole protein EV content only, but not in deiminated form. Our findings emphasize the putative differences in cell communication mediated by EV protein versus post-translationally deiminated protein cargo (the “EV-citrullinome”), providing novel insights into EV-mediated communication in halibut serum. Our findings furthermore contribute to current understanding of EV signatures across the phylogeny tree, with the potential for biomarker development and EV “fingerprinting” for the assessment of animal health.

## Figures and Tables

**Table 1:** Deiminated proteins in serum extracellular vesicles (EVs) of halibut (*Hippoglossus hippoglossus* L), as identified by F95-enrichment in conjunction with LC-MS/MS analysis. Deiminated proteins were isolated from serum-EVs from a pool of *n* = 4 fish, using immunoprecipitation with the pan-deimination F95 antibody. The resulting F95-enriched eluate was then analysed by LC-MS/MS and peak list files submitted to Mascot, using the Teleost UniProt database. Peptide sequence hits are listed, showing the number of sequences for protein hits and total score. Species hit names are indicated. In the case of uncharacterised protein ID, proteins matching the same set of peptides are indicated in brackets. Protein hits highlighted in pink (*) are specific to the F95 enriched EV fraction only. Protein names are written in bold. A full list of protein sequence hits and peptides is further provided in Supplementary Table S1.

Protein ID	Species Name	Matches	Total Score
**Protein Name**	Common Name	(Sequences)	(*p* < 0.05) ^ⱡ^
A0A6J2W3P0_CHACN	*Chanos chanos*	16 (13)	538
**Uncharacterised protein (histone H3-like)**	Milkfish		
A0A672ZYE0_9TELE	*Sphaeramia orbicularis*	12 (9)	451
**Uncharacterised protein**	Orbiculate cardinalfish		
A0A0A1G3Q1_9TELE	*Oxyeleotris marmorata*	10 (10)	427
**Beta-actin**	Marble goby		
A0A3P8Y5X6_ESOLU	*Esox Lucius*	26 (8)	356
**IF rod domain-containing protein**	Northern pike		
* W5ZLY1_9TELE	*Campylomormyrus compressirostris*	8 (8)	336
**Cytoplasmic 2 actin**	Elephantfish		
A0A3B4ZTX8_9TELE	*Stegastes partitus*	8 (7)	324
**Uncharacterized protein (NTR domain-containing protein; Complement component C3)**	Bicolour damselfish		
A0A3B4THR8_SERDU	*Seriola dumerili*	9 (8)	299
**Uncharacterized protein (NTR domain-containing protein; anaphylatoxin-like, Complement component C3)**	Greater amberjack		
A0A6G0HQ07_LARCR	*Larimichthys crocea*	8 (6)	281
**Histone H4**	Yellow croaker		
A0A3Q3IVX9_MONAL	*Monopterus albus*	8 (7)	276
**Uncharacterized protein (Complement C3)**	Asian swamp eel		
A0A3P9BEG5_9CICH	*Maylandia zebra*	6 (6)	273
**Uncharacterized protein (Anaphylatoxin-like, complement C3)**	Zebra mbuna		
A0A484CCU5_PERFV	*Perca flavescens*	8 (7)	271
**Uncharacterized protein (complement C3)**	Yellow perch		
A5JV31_HIPHI	*Hippoglossus hippoglossus*	7 (7)	261
**Phosvitin**	Atlantic halibut		
* A0A087XQB5_POEFO	*Poecilia formosa*	6 (5)	256
**Tubulin alpha chain**	Amazon molly		
A0A6F9CZC7_9TELE	*Coregonus sp. ‘balchen’*	5 (4)	251
**Uncharacterized protein (tubulin alpha-chain)**	Whitefish, salmonidae		
* Q1RLR3_DANRE	*Danio rerio*	8 (5)	237
**Keratin 93**	Zebrafish		
A0A1S5XZE7_9TELE	*Lipogramma levinsoni*	7 (7)	231
**Histone H3**	Hourglass basslet		
A3F5V1_ORENI	*Oreochromis niloticus*	7 (7)	222
**Beta actin (Fragment)**	Nile tilapia		
A0A5N5KJN7_PANHP	*Pangasianodon hypophthalmus*	6 (4)	185
**IF rod domain-containing protein**	Iridescent shark		
A0A4W6CP97_LATCA	*Lates calcarifer*	5 (4)	179
**Uncharacterized protein (Alpha-2-macroglobulin)**	Barramundi/Asian sea bass		
* H2MSJ5_ORYLA	*Oryzias latipes*	5 (4)	159
**Uncharacterized protein**	Medaka/Japanese rice fish		
A0A060WDP8_ONCMY	*Oncorhynchus mykiss*	3 (3)	136
**Elongation factor 1-alpha**	Rainbow trout		
A0A671UYU7_SPAAU	*Sparus aurata*	3 (1)	117
**Uncharacterized protein (A2M_recep domain-containing protein)**	Gilt-head bream		
G3Q4A0_GASAC	*Gasterosteus aculeatus*	2 (2)	116
**Fibrinogen beta chain**	Three-spined stickleback		
A0A0F8AH88_LARCR	*Larimichthys crocea*	2 (2)	107
**Ig heavy chain V region 5A**	Yellow croaker		
A0A4W6FLR7_LATCA	*Lates calcarifer*	3 (3)	104
**Uncharacterized protein (NTR domain-containing protein; anaphylatoxin like; A2M_N_2 domain-containing; complement C5)**	Barramundi/Asian sea bass		
A0A4Z2B138_9TELE	*Takifugu bimaculatus*	3 (3)	99
**Anaphylatoxin-like domain-containing protein**	Pufferfish		
Q4KVK3_HIPHI	*Hippoglossus hippoglossus*	2 (2)	94
**Complement component c3 (Fragment)**	Atlantic halibut		
* A0A5J5C7F1_9PERO	*Etheostoma spectabile*	2 (2)	94
**Uncharacterized protein (Fragment)**	Orangethroat darter		
* A0A0P7WL38_SCLFO	*Scleropages formosus*	4 (2)	93
**Trypsin-3-like**	Asian arowana		
Q5DVG8_PLAFE	*Platichthys flesus*	3 (2)	84
**Apolipoprotein AI**	European flounder		
A0A0F8ABH4_LARCR	*Larimichthys crocea*	3 (1)	82
**Granzyme B(G,H)**	Yellow croaker		
A0A484D989_PERFV	*Perca flavescens*	3 (2)	71
**Peptidase S1 domain-containing protein**	Yellow perch		
* A0A5N5Q536_PANHP	*Pangasianodon hypophthalmus*	2 (2)	70
**Centrosomal protein of 162 kDa**	Iridescent shark		
* A0A0P7UEW6_SCLFO	*Scleropages formosus*	1 (1)	69
**2-phospho-D-glycerate hydro-lyase**	Asian arowana		
A0A060YWU0_ONCMY	*Oncorhynchus mykiss*	4 (2)	68
**Peptidase S1 domain-containing protein**	Rainbow trout		
* A0A1A7WRH6_9TELE	*Iconisemion striatum*	2 (2)	64
**Integrin beta**	Killifish		
A0A3B5M528_9TELE	*Xiphophorus couchianus*	1 (1)	64
**Serotransferrin**	Monterrey platyfish		
A0A060Z3N3_ONCMY	*Oncorhynchus mykiss*	2 (2)	63
**Ig-like domain-containing protein**	Rainbow trout		
A0A060W543_ONCMY	*Oncorhynchus mykiss*	2 (2)	62
**Histone H2A**	Rainbow trout		
A0A0R4IU44_DANRE	*Danio rerio*	1 (1)	61
**Inter-alpha-trypsin inhibitor heavy chain 3b**	Zebrafish		
HV05_CARAU	*Carassius auratus*	2 (1)	60
**Ig heavy chain V region 5A**	Goldfish		
* A0A060XD44_ONCMY	*Oncorhynchus mykiss*	4 (2)	60
**Uncharacterized protein**	Rainbow trout		
A0A4W5L5T6_9TELE	*Hucho hucho*	1 (1)	57
**Thioredoxin**	Danube salmon		
* A0A3Q3LZB0_9TELE	*Mastacembelus armatus*	1 (1)	57
**Uncharacterized protein**	Zig-zag eel/Spiny eel		
* A0A5J5DS23_9PERO	*Etheostoma spectabile*	1 (1)	57
**Uncharacterized protein**	Orangethroat darter		
* A0A3B3QST7_9TELE	*Paramormyrops kingsleyae*	1 (1)	55
**Uncharacterized protein**	Elephantfish		
* A0A0E9RVI6_ANGAN	*Anguilla Anguilla*	1 (1)	53
**Uncharacterized protein**	European eel		
* A0A3Q3SSB4_9TELE	*Mastacembelus armatus*	1 (1)	53
* **Myosin_tail_1 domain-containing protein**	Zig-zag eel/Spiny eel		

^ⱡ^ Ions score is −10*Log(P), where P is the probability that the observed match is a random event. Individual ions scores > 53 indicate identity or extensive homology (*p* < 0.05). Protein scores are derived from ions scores as a non-probabilistic basis for ranking protein hits.

**Table 2:** Total protein cargo in serum-EVs of halibut (*Hippoglossus hippoglossus* L), as identified by LC-MS/MS analysis from serum-EVs isolated from a pool of sera from *n* = 4 fish. Peak list files were submitted to Mascot, using the Teleost UniProt database. Peptide sequence hits are listed, showing the number of sequences for protein hits and total score. Species hit names are indicated. In the case of uncharacterised protein ID, proteins matching the same set of peptides are indicated in brackets. Protein hits highlighted in blue (*) were not identified in the F95 enriched fraction. Protein names are written in bold. A full list of protein sequence hits and peptides is further provided in Supplementary Table S2.

Protein ID	Species Name	Matches	Total Score
Protein Name	Common Name	(Sequences)	(*p* < 0.05) ^ⱡ^
A5JV31_HIPHI	*Hippoglossus hippoglossus*	145 (56)	3616
**Phosvitin**	Atlantic halibut		
A5JV30_HIPHI	*Hippoglossus hippoglossus*	90 (52)	3303
**Phosvitin**	Atlantic halibut		
Q4KVK3_HIPHI	*Hippoglossus hippoglossus*	69 (25)	1690
**Complement component c3 (fragment)**	Atlantic halibut		
A0A2U9BPE5_SCOMX	*Scophthalmus maximus*	89 (24)	1426
**Complement component C3 isoform 2**	Turbot		
A0A3B4THR8_SERDU	*Seriola dumerili*	79 (22)	1269
**Uncharacterized protein (NTR domain-containing protein, Complement C3-like, A2M_recep domain-containing protein)**	Greater amberjack		
A0A3B4TYC3_SERDU	*Seriola dumerili*	65 (21)	1250
**NTR domain-containing protein**	Greater amberjack		
Q9PTY1_PAROL	*Paralichthys olivaceus*	70 (22)	1176
**Complement component C3**	Olive flounder		
G4WAB7_EPICO	*Epinephelus coioides*	57 (20)	1145
**Complement component c3**	Orange-spotted grouper		
A0A3P9BEG5_9CICH	*Maylandia zebra*	66 (19)	1120
**Uncharacterized protein (Anaphylatoxin-like domain-containing protein; C3a)**	Zebra mbuna		
* A0A669BPJ4_ORENI	*Oreochromis niloticus*	71 (18)	1097
**Uncharacterized protein**	Nile tilapia		
A0A671YHA0_SPAAU	*Sparus aurata*	45 (17)	904
**Uncharacterized protein (C3)**	Gilt-head bream		
* A0A6A5FQW4_PERFL	*Perca fluviatilis*	57 (16)	885
**Uncharacterized protein**	European perch		
A0A484CCU5_PERFV	*Perca flavescens*	56 (17)	879
**Uncharacterized protein (Anaphylatoxin-like domain-containing protein)**	Yellow perch		
F8R8R1_DICLA	*Dicentrarchus labrax*	59 (15)	871
**Complement component c3-2**	European bass		
A0A484DL37_PERFV	*Perca flavescens*	42 (15)	784
**Anaphylatoxin-like domain-containing protein**	Yellow perch		
A0A4W6E087_LATCA	*Lates calcarifer*	43 (15)	744
**Complement component c3a, duplicate 5**	Barramundi/Asian sea bass		
A0A6A5FJW4_PERFL	*Perca fluviatilis*	14 (12)	600
**Uncharacterized protein (Integrase catalytic domain-containing protein, Alpha-2-macroglobulin-like)**	European perch		
A0A2P9DTV2_SOLSE	*Solea senegalensis*	16 (10)	594
**Phosvitin**	Senegalese sole		
Q6QZI2_PSEAM	*Pseudopleuronectes americanus*	37 (9)	574
**Complement component C3 (Fragment)**	Winter flounder		
A0A3Q1ID66_ANATE	*Anabas testudineus*	25 (9)	569
**Phosvitin**	Climbing perch		
* A0A4W6F6V9_LATCA	*Lates calcarifer*	15 (9)	549
**Apolipoprotein Bb, tandem duplicate 2**	Barramundi/Asian sea bass		
A0A6G1PAV1_9TELE	*Channa argus*	38 (10)	540
**Complement C3 Complement C3 beta chain Complement C3 alpha chain**	Northern snakehead		
* A0A4P8JD10_9TELE	*Lateolabrax maculatus*	13 (9)	532
**Apolipoprotein Bb.1**	Spotted sea bass		
* A0A6A5DT05_PERFL	*Perca fluviatilis*	16 (9)	529
**Vitellogenin domain-containing protein**	European perch		
A0A673IJP2_9TELE	*Sinocyclocheilus rhinocerous*	48 (12)	528
**IF rod domain-containing protein**	Sinocyclocheilus cavefish (Cyprinid)		
A0A4W6CMC4_LATCA	*Lates calcarifer*	14 (11)	525
**Uncharacterized protein (Alpha-2-macroglobulin)**	Barramundi/Asian sea bass		
A0A3P8Y5X6_ESOLU	*Esox Lucius*	51 (11)	499
**IF rod domain-containing protein**	Northern pike		
A0A6G1PQL3_9TELE	*Channa argus*	12 (10)	497
**Alpha-2-macroglobulin**	Northern snakehead		
A0A6A4SX26_SCOMX	*Scophthalmus maximus*	51 (11)	463
**IF rod domain-containing protein**	Turbot		
Q5DVG8_PLAFE	*Platichthys flesus*	26 (9)	453
**Apolipoprotein AI**	European flounder		
* A0A3B4T6U1_SERDU	*Seriola dumerili*	12 (9)	440
**Vitellogenin domain-containing protein**	Greater amberjack		
A0A665VQL3_ECHNA	*Echeneis naucrates*	9 (8)	409
**Uncharacterized protein (A2M_N_2 domain-containing protein)**	Live sharksucker		
* A0A2U9D044_SCOMX	*Scophthalmus maximus*	14 (8)	407
**Putative apolipoprotein B-100-like isoform 2**	Turbot		
* Q9PVW6_PAROL	*Paralichthys olivaceus*	14 (7)	403
**Complement component C9**	Olive flounder		
A0A4W6FLR7_LATCA	*Lates calcarifer*	10 (8)	386
**Uncharacterized protein (Anaphylatoxin-like domain-containing, A2M_N_2 domain containing protein, NTR domain containing protein, Complement C5)**	Barramundi/Asian sea bass		
A0A4W6CP97_LATCA	*Lates calcarifer*	17 (7)	362
**Uncharacterized protein (A2M_recep domain-containing protein, TED_complement domain-containing protein)**	Barramundi/Asian sea bass		
* A0A3P8RR96_AMPPE	*Amphiprion percula*	12 (5)	353
**Complement component C9**	Orange clownfish		
A0A3Q1HZ43_ANATE	*Anabas testudineus*	13 (9)	336
**Uncharacterized protein (Inter-alpha-trypsin inhibitor, VIT domain-containing protein)**	Climbing perch		
* A0A3Q1H6Y9_ANATE	*Anabas testudineus*	8 (8)	336
**Complement component 8 subunit beta**	Climbing perch		
A0A6G1PI27_9TELE	*Channa argus*	13 (7)	324
**Inter-alpha-trypsin inhibitor heavy chain H3**	Northern snakehead		
A0A6A5FLM2_PERFL	*Perca fluviatilis*	10 (6)	323
**Uncharacterized protein (alpha-2-macroglobulin-like, A2M_recep domain-containing protein)**	European perch		
A0A6A5FFR2_PERFL	*Perca fluviatilis*	15 (7)	323
**Anaphylatoxin-like domain-containing protein**	European perch		
A0A484DIJ5_PERFV	*Perca flavescens*	11 (7)	321
**Uncharacterized protein (Alpha-2-macroglobulin)**	Yellow perch		
A0A6A5FE70_PERFL	*Perca fluviatilis*	11 (7)	318
**Uncharacterized protein (A2M_recep domain-containing, MG2 domain-containing protein)**	European perch		
A0A6J2W3P0_CHACN	*Chanos chanos*	8 (7)	312
**uncharacterized protein LOC115819396 (Histone H4, Histone H3, Histone H2B)**	Milkfish		
* A0A665V532_ECHNA	*Echeneis naucrates*	8 (6)	310
**Plasminogen**	Live sharksucker		
A0A3Q3L7G2_9TELE	*Mastacembelus armatus*	6 (6)	308
**Complement component c3b, tandem duplicate 2**	Zig-zag eel/Spiny eel		
* CO8B_PAROL	*Paralichthys olivaceus*	5 (5)	304
**Complement component C8 beta chain**	Olive flounder		
A0A671PIL3_9TELE	*Sinocyclocheilus anshuiensis*	17 (6)	301
**IF rod domain-containing protein**	Sinocyclocheilus cavefish (Cyprinoid)		
* A0A3Q3E5X5_9LABR	*Labrus bergylta*	7 (4)	298
**Uncharacterized protein (C1q domain-containing protein)**	Ballan wrasse		
* A0A3Q0S0V4_AMPCI	*Amphilophus citrinellus*	18 (5)	292
**Uncharacterized protein**	Midas cichlid		
A0A6A4SU52_SCOMX	*Scophthalmus maximus*	7 (7)	291
**Uncharacterized protein (Complement component c3b)**	Turbot		
* A0A3P8TA20_AMPPE	*Amphiprion percula*	11 (7)	290
**Zgc:112265**	Orange clownfish		
A0A096MDQ7_POEFO	*Poecilia formosa*	11 (6)	288
**Phosvitin**	Amazon molly		
Q5XVQ2_FUNHE	*Fundulus heteroclitus*	17 (5)	288
**Apolipoprotein A1 (Fragment)**	Atlantic killifish, mud minnow		
* Q6QZI9_PSEAM	*Pseudopleuronectes americanus*	12 (5)	284
**Complement component C9 (Fragment)**	Winter flounder		
* A0A4U5UPP9_COLLU	*Collichthys lucidus*	7 (5)	283
**Apolipoprotein B-100**	(Big head croaker)		
A0A3Q1EMN2_9TELE	*Acanthochromis polyacanthus*	8 (5)	280
**Uncharacterized protein (beta actin, actin cytoplasmic-1)**	Spiny chromis damselfish		
* A0A6J2P874_COTGO	*Cottoperca gobio*	7 (5)	280
**plasminogen**	Channel bull blenny		
A0A3B4VID4_SERDU	*Seriola dumerili*	9 (5)	279
**Uncharacterized protein (MG2 domain-containing protein)**	Greater amberjack		
* A0A3Q3M9S2_9TELE	*Mastacembelus armatus*	12 (4)	278
**Uncharacterized protein**	Zig-zag eel/Spiny eel		
W5ZMG9_9TELE	*Campylomormyrus compressirostris*	7 (4)	267
**Cytoplasmic 1 actin**	Elephantfish		
A0A553Q7M4_9TELE	*Danionella translucida*	6 (6)	262
**Uncharacterized protein (Histone H2A, H2B putative, H3)**	Micro glassfish (Cyprinid)		
A0A3Q1H0X2_ANATE	*Anabas testudineus*	5 (5)	260
**Complement component c3b, tandem duplicate 2**	Climbing perch		
* A0A6A4SHP5_SCOMX	*Scophthalmus maximus*	12 (5)	258
**Uncharacterized protein**	Turbot		
* G3NNM8_GASAC	*Gasterosteus aculeatus*	6 (6)	256
**Uncharacterized protein**	Three-spined stickleback		
* A0A0P7YVM9_SCLFO	*Scleropages formosus*	10 (5)	251
**Keratin, type I cytoskeletal 13-like**	Asian arowana		
* A0A6A4SWR2_SCOMX	*Scophthalmus maximus*	7 (6)	251
**EGF-like domain-containing protein**	Turbot		
A0A2U9B3I5_SCOMX	*Scophthalmus maximus*	13 (6)	247
**Alpha-2-macroglobulin**	Turbot		
A0A4Z2BCD9_9TELE	*Takifugu bimaculatus*	6 (5)	242
**Uncharacterized protein**	Pufferfish		
**(Complement C5 C3 and PZP-like alpha-2-macroglobulin domain-containing protein)**			
A0A671TD78_SPAAU	*Sparus aurata*	5 (5)	238
**Complement component c3b, tandem duplicate 2**	Gilt-head bream		
* A0A0A0QKL5_OPLFA	*Oplegnathus fasciatus*	6 (5)	234
**Complement component 4**	Striped beakfish		
* A0A6A4RUD7_SCOMX	*Scophthalmus maximus*	6 (6)	233
**Vitellogenin domain-containing protein**	Turbot		
A0A672YA60_9TELE	*Sphaeramia orbicularis*	7 (6)	232
**Uncharacterized protein (inter-alpha-trypsin inhibitor heavy chain)**	Orbiculate cardinalfish		
* A0A672JL95_SALFA	*Salarias fasciatus*	5 (5)	232
**Uncharacterized protein (complement C7)**	Lawnmower blenny		
* A0A3B4Y8X6_SERLL	*Seriola lalandi*	10 (4)	231
**Uncharacterized protein (Hephaestin-like protein 1, Desmoglein-2)**	Yellowtail amberjack		
* A0A3B4UHS2_SERDU	*Seriola dumerili*	4 (4)	229
**Uncharacterized protein**	Greater amberjack		
* A0A087YMZ0_POEFO	*Poecilia formosa*	11 (6)	229
**Uncharacterized protein (Ceruloplasmin)**	Amazon molly		
A0A3Q4G4S3_NEOBR	*Neolamprologus brichardi*	11 (5)	229
**Uncharacterized protein (NTR domain-containing protein)**	Lyretail cichlid		
* A0A3Q1EBE7_9TELE	*Acanthochromis polyacanthus*	4 (4)	228
**Vitellogenin domain-containing protein**	Spiny chromis damselfish		
A0A3P9A8D3_ESOLU	*Esox Lucius*	8 (4)	218
**Uncharacterized protein (Alpha-2-macroglobulin, A2M_recep domain-containing)**	Northern pike		
* A0A3P8WZ01_CYNSE	*Cynoglossus semilaevis*	7 (4)	217
**Vitellogenin domain-containing protein**	Tongue sole		
* A0A3B4F9T0_9CICH	*Pundamilia nyererei*	3 (3)	215
**Carboxypeptidase Q**	Cichlid		
* A0A6J2QSS9_COTGO	*Cottoperca gobio*	3 (2)	209
**complement component C9**	Channel bull blenny		
* A0A672GNQ4_SALFA	*Salarias fasciatus*	7 (4)	208
**Vitellogenin domain-containing protein**	Lawnmower blenny		
* A0A3B4ULR2_SERDU	*Seriola dumerili*	9 (5)	207
**Zgc:112265**	Greater amberjack		
A0A3B4THN2_SERDU	*Seriola dumerili*	4 (4)	205
**Fibrinogen beta chain**	Greater amberjack		
* A0A2U9BK85_SCOMX	*Scophthalmus maximus*	3 (3)	203
**Putative complement component C8 alpha chain**	Turbot		
* G8DP14_PLAFE	*Platichthys flesus*	4 (4)	201
**Beta 1-globin**	European flounder		
* A0A0F8C5A6_LARCR	*Larimichthys crocea*	6 (5)	200
**Antithrombin-III**	Yellow croaker		
* A0A2U9CEJ2_SCOMX	*Scophthalmus maximus*	4 (4)	200
**Complement component 7**	Turbot		
A0A5C6MX12_9TELE	*Takifugu flavidus*	17 (5)	196
**Complement C3**	Yellowbelly pufferfish		
* Q6QZI5_PSEAM	*Pseudopleuronectes americanus*	4 (4)	194
**Complement component C8 beta chain**	Winter flounder		
* A0A3B3BJ38_ORYME	*Oryzias melastigma*	7 (4)	192
**Vitellogenin domain-containing protein**	Marine medaka		
* A0A6J2S534_COTGO	*Cottoperca gobio*	5 (5)	190
**apolipoprotein B-100**	Channel bull blenny		
* A0A3Q1JFY5_ANATE	*Anabas testudineus*	5 (3)	187
**Uncharacterized protein (ceruloplasmin)**	Climbing perch		
A0A672I1M9_SALFA	*Salarias fasciatus*	6 (4)	186
**Uncharacterized protein (Inter-alpha-trypsin inhibitor heavy chain, VIT domain-containing protein)**	Lawnmower blenny		
A0A3B5AT07_9TELE	*Stegastes partitus*	7 (4)	185
**IF rod domain-containing protein**	Bicolour damselfish		
* A0A4Z2CEC7_9TELE	*Takifugu bimaculatus*	4 (4)	183
**Uncharacterized protein (complement C4)**	Pufferfish		
* A0A3Q3II57_MONAL	*Monopterus albus*	5 (4)	183
**Uncharacterized protein**	Asian swamp eel		
* A0A3Q3FIH8_KRYMA	*Kryptolebias marmoratus*	7 (4)	180
**Uncharacterized protein**	Mangrove rivulus(killilfish)		
* A0A2U9AYP3_SCOMX	*Scophthalmus maximus*	5 (3)	177
**Complement component 4**	Turbot		
* A0A6J2RDF1_COTGO	*Cottoperca gobio*	4 (4)	176
**complement C4-B-like**	Channel bull blenny		
A0A4W6ERJ2_LATCA	*Lates calcarifer*	5 (4)	173
**Fibrinogen gamma chain**	Barramundi/Asian sea bass		
* A0A2I4C034_9TELE	*Austrofundulus limnaeus*	3 (3)	167
**collagen alpha-1(XII) chain**	Killifish		
* A0A6I9PPD4_9TELE	*Notothenia coriiceps*	4 (4)	167
**complement C4-like**	Black rockcod/Antarctic yellowbelly rockcod		
* H3BWT7_TETNG	*Tetraodon nigroviridis*	5 (3)	163
**Ceruloplasmin**	Green spotted puffer		
* Q4SXM5_TETNG	*Tetraodon nigroviridis*	5 (4)	160
**Chromosome 12 SCAF12357, whole genome shotgun sequence**	Green spotted puffer		
A0A1A8F2V0_9TELE	*Nothobranchius korthausae*	5 (2)	160
**Uncharacterized protein (Alpha2-macroglobulin)**	Killifish		
* A0A3B5BD88_9TELE	*Stegastes partitus*	4 (4)	159
**Vitellogenin domain-containing protein**	Bicolour damselfish		
A0A6G1QB31_9TELE	*Channa argus*	9 (2)	159
**Serotransferrin**	Northern snakehead		
* A0A060WU48_ONCMY	*Oncorhynchus mykiss*	2 (2)	157
**Uncharacterized protein (Desmoplakin)**	Rainbow trout		
*A0A3Q3FAE5_9LABR	*Labrus bergylta*	4 (3)	155
**Complement component 8 subunit beta**	Ballan wrasse		
A0A6J2Q526_COTGO	*Cottoperca gobio*	4 (3)	155
**fibrinogen gamma chain**	Channel bull blenny		
* A0A3B4UV22_SERDU	*Seriola dumerili*	6 (4)	154
**Antithrombin-III**	Greater amberjack		
* A0A3Q2QAA5_FUNHE	*Fundulus heteroclitus*	4 (3)	154
**Uncharacterized protein**	Atlantic killifish, mud minnow		
* A0A6J2P7B9_COTGO	*Cottoperca gobio*	3 (2)	153
**apolipoprotein B-100-like**	Channel bull blenny		
* A0A484D0P7_PERFV	*Perca flavescens*	6 (5)	153
**Uncharacterized protein (ceruloplasmin)**	Yellow perch		
* A0A3B4TA89_SERDU	*Seriola dumerili*	3 (3)	149
**Uncharacterized protein**	Greater amberjack		
* A0A673XMC1_SALTR	*Salmo trutta*	3 (3)	148
**Uncharacterized protein (complement C4, C4-B)**	Brown trout		
F8U8N8_CHELB	*Chelon labrosus*	4 (3)	146
**Alpha 2 macroglobulin (fragment)**	Thicklip grey mullet		
F2Y9S5_MORSA	*Morone saxatilis*	3 (3)	145
**Phosvitin**	Striped bass		
* A0A3P9Q7U6_POERE	*Poecilia reticulate*	4 (4)	144
**Complement component C9**	Guppy		
A0A0F8AH88_LARCR	*Larimichthys crocea*	9 (3)	143
**Ig heavy chain V region 5A**	Yellow croaker		
* A0A667Y3E0_9TELE	*Myripristis murdjan*	6 (3)	142
**Vitellogenin domain-containing protein**	Blacktipped soldierfish		
* A0A672QEF7_SINGR	*Sinocyclocheilus graham*	8 (4)	141
**Uncharacterized protein**	Golden-line barbell		
* A0A3B5B7I8_9TELE	*Stegastes partitus*	5 (4)	141
**Antithrombin-III**	Bicolour damselfish		
* A0A0B6VKQ1_ORYCL	*Oryzias celebensis*	3 (3)	139
**B5 protein**	Celebes medaka		
* A0A671TKG8_SPAAU	*Sparus aurata*	4 (2)	138
**Uncharacterized protein**	Gilt-head bream		
* A0A4P8JCG0_9TELE	*Lateolabrax maculatus*	3 (2)	136
**Apolipoprotein Bb.2**	Spotted sea bass		
* A0A3B4FS46_9CICH	*Pundamilia nyererei*	4 (3)	132
**IGv domain-containing protein**	Cichlid		
A0A3P9H4Z3_ORYLA	*Oryzias latipes*	9 (3)	132
**Uncharacterized protein (A2M_N_2 domain-containing protein, anaphylatoxin-like domain)**	Medaka/Japanese rice fish		
A0A0F8AKQ4_LARCR	*Larimichthys crocea*	5 (3)	131
**Alpha-2-macroglobulin**	Yellow croaker		
A0A3B4TIN1_SERDU	*Seriola dumerili*	3 (3)	130
**Phosvitin**	Greater amberjack		
B6RUP0_ORYDN	*Oryzias dancena*	4 (3)	129
**Beta-actin (Fragment)**	Indian ricefish		
* A0A484CD54_PERFV	*Perca flavescens*	3 (3)	129
**Uncharacterized protein (Complement C7)**	Yellow perch		
A0A3Q4FXR7_NEOBR	*Neolamprologus brichardi*	4 (3)	128
**Ig-like domain-containing protein**	Lyretail cichlid		
Q5SET8_9TELE	*Bembras japonica*	3 (3)	128
**Histone H3 (Fragment)**	Red flathead		
A0A3Q1IXI9_ANATE	*Anabas testudineus*	3 (3)	128
**Uncharacterized protein (A2M_recep domain-containing protein)**	Climbing perch		
* A0A4Z2H8W0_9TELE	*Liparis tanakae*	2 (2)	126
**Biotinidase**	Tanaka’s snailfish		
A0A6G1PSN0_9TELE	*Channa argus*	6 (5)	126
**Alpha-2-macroglobulin**	Northern snakehead		
A0A669DKF1_ORENI	*Oreochromis niloticus*	4 (3)	125
**Uncharacterized protein (Ig-like domain-containing protein)**	Nile tilapia		
A0A3B1JCF6_ASTMX	*Astyanax mexicanus*	6 (3)	123
**IF rod domain-containing protein**	Mexican tetra/blind cave fish		
* A0A3Q2YHX2_HIPCM	*Hippocampus comes*	3 (3)	122
**Complement component 8 subunit beta**	Tiger tail seahorse		
A0A3Q3IC70_MONAL	*Monopterus albus*	1 (1)	121
**Ig-like domain-containing protein**	Asian swamp eel		
* A0A0F8AI97_LARCR	*Larimichthys crocea*	2 (2)	121
**Collagenase 3**	Yellow croaker		
A0A6J2PEG5_COTGO	*Cottoperca gobio*	2 (2)	120
**complement C5-like**	Channel bull blenny		
A0A6A4TFM7_SCOMX	*Scophthalmus maximus*	3 (2)	119
**Ig-like domain-containing protein**	Turbot		
* A0A3Q0R4Z0_AMPCI	*Amphilophus citrinellus*	5 (3)	119
**Complement component C9**	Midas cichlid		
A0A6I9NNH1_9TELE	*Notothenia coriiceps*	3 (2)	118
**inter-alpha-trypsin inhibitor heavy chain H2**	Black rockcod/Antarctic yellowbelly rockcod		
A0A437D6V7_ORYJA	*Oryzias javanicus*	2 (2)	114
**Chitinase**	Javanese ricefish		
A0A3Q3EEY5_9LABR	*Labrus bergylta*	3 (3)	113
**Fibrinogen C-terminal domain-containing protein**	Ballan wrasse		
* A0A1S3SMN1_SALSA	*Salmo salar*	1 (1)	111
**cathepsin L1-like**	Atlantic salmon		
* A0A3Q3IZL2_MONAL	*Monopterus albus*	4 (3)	111
**Uncharacterized protein**	Asian swamp eel		
* A0A3P8YF02_ESOLU	*Esox Lucius*	6 (3)	109
**Vitellogenin domain-containing protein**	Northern pike		
* A0A3B3CJZ7_ORYME	*Oryzias melastigma*	3 (2)	109
**Complement 4B (Chido blood group)**	Marine medaka		
* A0A2U9CVZ8_SCOMX	*Scophthalmus maximus*	2 (2)	108
**Putative complement component C8 gamma chain**	Turbot		
A0A3Q3RJX0_9TELE	*Mastacembelus armatus*	2 (1)	108
**Ig-like domain-containing protein**	Zig-zag eel/Spiny eel		
* A0A3Q4HZS4_NEOBR	*Neolamprologus brichardi*	3 (3)	108
**Uncharacterized protein (Ceruloplasmin)**	Lyretail cichlid		
A0A6G1PYT4_9TELE	*Channa argus*	4 (3)	108
**Complement C5 C3 and PZP-like alpha-2-macroglobulin domain-containing protein 4**	Northern snakehead		
* A0A2U9AV20_SCOMX	*Scophthalmus maximus*	2 (2)	107
**Prothrombin**	Turbot		
A0A4W6EWH0_LATCA	*Lates calcarifer*	3 (3)	107
**Peptidase S1 domain-containing protein**	Barramundi/Asian sea bass		
* H3C6P0_TETNG	*Tetraodon nigroviridis*	2 (2)	106
**Plasminogen**	Green spotted puffer		
A0A3P8SDE5_AMPPE	*Amphiprion percula*	14 (2)	105
**Serotransferrin**	Orange clownfish		
A0A3B4BP10_PYGNA	*Pygocentrus nattereri*	10 (2)	105
**Uncharacterized protein**	Red-bellied piranha		
* A0A3B4TQB5_SERDU	*Seriola dumerili*	1 (1)	105
**SERPIN domain-containing protein**	Greater amberjack		
* D5A7I1_DICLA	*Dicentrarchus labrax*	4 (2)	104
**Hemopexin**	European bass		
* A0A2U9CU10_SCOMX	*Scophthalmus maximus*	3 (3)	103
**Putative insulin-like growth factor-binding protein complex acid labile subunit**	Turbot		
* A0A6J2PA80_COTGO	*Cottoperca gobio*	3 (3)	103
**histone H2B 1/2-like**	Channel bull blenny		
* A0A3P8SSL4_AMPPE	*Amphiprion percula*	2 (2)	102
**Uncharacterized protein (Ig-like domain-containing protein, Nattectin)**	Orange clownfish		
* G3NN36_GASAC	*Gasterosteus aculeatus*	4 (3)	99
**Uncharacterized protein**	Three-spined stickleback		
A0A4W4FLR8_ELEEL	*Electrophorus electricus*	3 (2)	99
**Fibrinogen beta chain**	Electric eel		
A0A671TDU8_SPAAU	*Sparus aurata*	2 (2)	97
**Ig-like domain-containing protein**	Gilt-head bream		
A0A6I9PPY0_9TELE	*Notothenia coriiceps*	2 (2)	96
**fibrinogen gamma chain**	Black rockcod/Antarctic yellowbelly rockcod		
A0A671TNW0_SPAAU	*Sparus aurata*	4 (3)	96
**Histone H3**	Gilt-head bream		
* A0A3B4XVK3_SERLL	*Seriola lalandi dorsalis*	2 (2)	96
**Vitellogenin domain-containing protein**	Yellowtail amberjack		
* A0A3Q3L1F9_9TELE	*Mastacembelus armatus*	1 (1)	95
**Complement component 1, r subcomponent**	Zig-zag eel/Spiny eel		
A0A1A8AN27_NOTFU	*Nothobranchius furzeri*	3 (3)	95
**Fibrinogen, gamma polypeptide**	turquoise killifish		
* A0A2D0QC28_ICTPU	*Ictalurus punctatus*	2 (2)	93
**Ig heavy chain Mem5-like**	Channel catfish		
A0A3P8R4C1_ASTCA	*Astatotilapia calliptera*	6 (2)	96
**Uncharacterized protein (Ig-like domain-containing protein)**	Eastern happy/eastern river bream		
A0A3B4H9E9_9CICH	*Pundamilia nyererei*	3 (2)	93
**Ig-like domain-containing protein**	Cichlid		
A0A3B4UNU3_SERDU	*Seriola dumerili*	4 (2)	93
**Ig-like domain-containing protein**	Greater amberjack		
* A0A060XWP2_ONCMY	*Oncorhynchus mykiss*		92
**SERPIN domain-containing protein**	Rainbow trout		
* A0A1A8CRV1_9TELE	*Nothobranchius kadleci*	8 (2)	91
**Uncharacterized protein**	Killifish		
* A0A2U9CFI3_SCOMX	*Scophthalmus maximus*	2 (2)	90
**Putative sushi domain-containing protein 2 isoform 2**	Turbot		
A0A5C6NS08_9TELE	*Takifugu flavidus*	5 (2)	90
**Ig heavy chain V region VH558 A1/A4**	Yellowbelly pufferfish		
* A0A4W4DXU4_ELEEL	*Electrophorus electricus*	3 (3)	89
**14_3_3 domain-containing protein**	Electric eel		
* A0A0F8B5M5_LARCR	*Larimichthys crocea*	1 (1)	88
**Catechol O-methyltransferase domain-containing protein 1**	Yellow croaker		
* A0A5N5KRW8_PANHP	*Pangasianodon hypophthalmus*	3 (3)	88
**Uncharacterized protein (pleckstrin homology domain-containing family)**	Iridescent shark		
* A0A5C6NRB2_9TELE	*Takifugu flavidus*	2 (2)	87
**Apolipoprotein B-100**	Yellowbelly pufferfish		
A0A2D0RGG9_ICTPU	*Ictalurus punctatus*	3 (2)	87
**catenin beta-1 isoform X3**	Channel catfish		
* A0A6I9P4Q9_9TELE	*Notothenia coriiceps*	1 (1)	86
**apolipoprotein B-100-like**	Black rockcod/Antarctic yellowbelly rockcod		
* A0A087XVJ8_POEFO	*Poecilia formosa*	2 (1)	86
**Uncharacterized protein (IGv domain-containing protein)**	Amazon molly		
* H1AB41_PLASA	*Platichthys stellatus*	4 (2)	85
**Lysozyme**	Starry flounder		
* A0A4P8JEC9_9TELE	*Lateolabrax maculatus*	2 (2)	84
**Apolipoprotein Ba**	Spotted sea bass		
A0A3Q4ACH4_MOLML	*Mola mola*	2 (2)	84
**Inter-alpha-trypsin inhibitor heavy chain 3**	Ocean sunfish		
* A0A484CC61_PERFV	*Perca flavescens*	3 (1)	84
**Uncharacterized protein (Hyaluronan-binding protein 2)**	Yellow perch		
A0A060Z3N3_ONCMY	*Oncorhynchus mykiss*	3 (2)	86
**Ig-like domain-containing protein**	Rainbow trout		
* A0A3B4ZU87_9TELE	*Stegastes partitus*	3 (2)	83
**Uncharacterized protein (complement factor H-like)**	Bicolour damselfish		
A0A3B3QDE5_9TELE	*Paramormyrops kingsleyae*	2 (1)	83
**Ig-like domain-containing protein**	Elephantfish		
A0A3B3CFL8_ORYME	*Oryzias melastigma*	5 (2)	83
**Ig-like domain-containing protein**	Marine medaka		
* A0A3Q3W6Q7_MOLML	*Mola mola*	2 (2)	82
**Sushi domain containing 2**	Ocean sunfish		
A0A4W5L5T6_9TELE	*Hucho hucho*	3 (2)	82
**Thioredoxin**	Danube salmon		
* G1DHP8_GOBRA	*Gobiocypris rarus*	2 (2)	81
**Vitellogenin (Fragment)**	Rare gudgeon/rare minnow		
* A0A3B3QP35_9TELE	*Paramormyrops kingsleyae*	3 (2)	80
**Uncharacterized protein**	Elephantfish		
* A0A3B4Z082_9TELE	*Stegastes partitus*	2 (2)	80
**Uncharacterized protein (complement C6)**	Bicolour damselfish		
A0A669CCK4_ORENI	*Oreochromis niloticus*	6 (2)	80
**Uncharacterized protein (Ig-like domain-containing protein)**	Nile tilapia		
* A0A484C6M0_PERFV	*Perca flavescens*	1 (1)	80
**Uncharacterized protein**	Yellow perch		
* A0A3P8U2B4_AMPPE	*Amphiprion percula*	4 (2)	80
**Keratin 98**	Orange clownfish		
* A0A060WHH8_ONCMY	*Oncorhynchus mykiss*	2 (2)	79
**Junction plakoglobin**	Rainbow trout		
A0A3B4ULY5_SERDU	*Seriola dumerili*	4 (2)	78
**Ig-like domain-containing protein**	Greater amberjack		
H3C0U1_TETNG	*Tetraodon nigroviridis*	3 (2)	77
**Ig-like domain-containing protein**	Green spotted puffer		
A0A087X4F8_POEFO	*Poecilia formosa*	1 (1)	77
**Uncharacterized protein (Ig-like domain-containing protein)**	Amazon molly		
A0A3P9IRN4_ORYLA	*Oryzias latipes*	2 (2)	77
**Ig-like domain-containing protein**	Medaka/Japanese rice fish		
A0A060W543_ONCMY	*Oncorhynchus mykiss*	2 (2)	77
**Histone H2A**	Rainbow trout		
A0A3B4UFJ1_SERDU	*Seriola dumerili*	2 (1)	75
**Ig-like domain-containing protein**	Greater amberjack		
A0A0F8ABH4_LARCR	*Larimichthys crocea*	5 (1)	75
**Granzyme B(G,H)**	Yellow croaker		
* A0A3B4UPX8_SERDU	*Seriola dumerili*	1 (1)	74
**Zona pellucida sperm-binding protein 3**	Greater amberjack		
* A0A3P8U813_AMPPE	*Amphiprion percula*	2 (2)	73
**Si:ch1073-416d2.3**	Orange clownfish		
* A0A3Q1KAD2_ANATE	*Anabas testudineus*	2 (2)	72
**SERPIN domain-containing protein**	Climbing perch		
* A0A4W5RID4_9TELE	*Hucho hucho*	2 (1)	71
**RRM domain-containing protein**	Danube salmon		
* A0A3Q2QNZ9_FUNHE	*Fundulus heteroclitus*	2 (2)	71
**Uncharacterized protein (Sushi domain containing 2)**	Atlantic killifish, mud minnow		
* A0A4W5LQ29_9TELE	*Hucho hucho*	8 (2)	70
**ATP-synt ab_N domain-containing protein**	Danube salmon		
A0A3Q2PS35_FUNHE	*Fundulus heteroclitus*	5 (2)	70
**Ig-like domain-containing protein**	Atlantic killifish, mud minnow		
* A0A6G1QID3_9TELE	*Channa argus*	2 (2)	70
**Complement component C6**	Northern snakehead		
* A0A3B3X986_9TELE	*Poecilia Mexicana*	1 (1)	70
**Uncharacterized protein (F-BAR domain-containing protein)**	Atlantic (shortfin) molly		
* A0A498LNY2_LABRO	*Labeo rohita*	6 (2)	70
**Retrotransposon-derived PEG10**	Rohu		
* A0A6G1PD67_9TELE	*Channa argus*	2 (2)	70
**Apoptosis-stimulating of p53 protein 2 Bcl2-binding protein**	Northern snakehead		
* A0A1S3L2W1_SALSA	*Salmo salar*	5 (2)	70
**FH2 domain-containing protein 1-like**	Atlantic salmon		
A0A3B3HM39_ORYLA	*Oryzias latipes*	1 (1)	69
**Ig-like domain-containing protein**	Medaka/Japanese rice fish		
* A0A3Q1HK94_ANATE	*Anabas testudineus*	6 (2)	69
**Protein-tyrosine-phosphatase**	Climbing perch		
A0A3Q3JUN7_MONAL	*Monopterus albus*	2 (2)	68
**IF rod domain-containing protein**	Asian swamp eel		
* A0A671X983_SPAAU	*Sparus aurata*	3 (2)	68
**Uncharacterized protein (Early endosome antigen 1, FYVE-type domain-containing protein)**	Gilt-head bream		
* A0A3B3DTR8_ORYME	*Oryzias melastigma*	3 (2)	68
**Uncharacterized protein**	Marine medaka		
* A0A3Q3XI23_MOLML	*Mola mola*	3 (2)	67
**Zgc:112265**	Ocean sunfish		
A0A671YT10_SPAAU	*Sparus aurata*	2 (2)	67
**Uncharacterized protein (Immunoglobulin like and fibronectin type III domain containing 1, tandem duplicate 2)**	Gilt-head bream		
* A0A3B5ACM2_9TELE	*Stegastes partitus*	6 (2)	66
**Uncharacterized protein**	Bicolour damselfish		
A0A3P9H0Y9_ORYLA	*Oryzias latipes*	2 (2)	65
**Ig-like domain-containing protein**	Medaka/Japanese rice fish		
* A0A5C6N3H2_9TELE	*Takifugu flavidus*	4 (2)	65
**Keratin, type I cytoskeletal 18**	Yellowbelly pufferfish		
* A0A3B5L5A5_9TELE	*Xiphophorus couchianus*	3 (2)	65
**Thyroid hormone receptor interactor 11**	Monterrey platyfish		
* Q2PZ29_SOLSE	*Solea senegalensis*	2 (1)	65
**Lysozyme**	Senegalese sole		
A0A667YBU1_9TELE	*Myripristis murdjan*	5 (2)	65
**Ig-like domain-containing protein**	Blacktipped soldierfish		
* A0A672GWK0_SALFA	*Salarias fasciatus*	2 (2)	64
**Uncharacterized protein (Complement factor B-like)**	Lawnmower blenny		
* A0A3B4CEW8_PYGNA	*Pygocentrus nattereri*	2 (2)	64
**Uncharacterized protein (Roundabout-like axon guidance receptor protein 2)**	Red-bellied piranha		
* A0A3B4EX20_9CICH	*Pundamilia nyererei*	6 (2)	64
**Uncharacterized protein (Apolipoprotein M)**	Cichlid		
* A0A2I4BMF1_9TELE	*Austrofundulus limnaeus*	1 (1)	63
**protein Z-dependent protease inhibitor-like**	Killifish		
* A0A3Q3EPX4_9LABR	*Labrus bergylta*	2 (2)	62
**Vitellogenin domain-containing protein**	Ballan wrasse		
* A0A3B4T5U4_SERDU	*Seriola dumerili*	3 (2)	62
**Uncharacterized protein (Myosin phosphatase Rho interacting protein)**	Greater amberjack		
A0A3B3T2D8_9TELE	*Paramormyrops kingsleyae*	1 (1)	62
**Ig-like domain-containing protein**	Elephantfish		
* A0A3Q1FWV1_9TELE	*Acanthochromis polyacanthus*	2 (2)	62
**Multidrug and toxin extrusion protein**	Spiny chromis damselfish		
A0A3B4YHZ5_SERLL	*Seriola lalandi dorsalis*	1 (1)	61
**IGv domain-containing protein**	Yellowtail amberjack		
* R4I5B0_EPICO	*Epinephelus coioides*	3 (2)	61
**Immmunoglobulin light chain**	Orange-spotted grouper		
* A0A3Q0R568_AMPCI	*Amphilophus citrinellus*	3 (2)	61
**FH2 domain containing 4**	Midas cichlid		
A0A3B4WXW5_SERLL	*Seriola lalandi dorsalis*	2 (2)	60
**Ig-like domain-containing protein**	Yellowtail amberjack		
G3PK20_GASAC	*Gasterosteus aculeatus*	3 (2)	60
**Serotransferrin**	Three-spined stickleback		
* A0A484DB45_PERFV	*Perca flavescens*	1 (1)	60
**Uncharacterized protein (Pentaxin)**	Yellow perch		
* A0A671SV95_9TELE	*Sinocyclocheilus anshuiensis*	2 (2)	60
**FERM domain-containing protein**	Sinocyclocheilus cavefish (Cyprinoid)		
A0A023REA6_9TELE	*Menidia estor*	1 (1)	60
**Elongation factor 1-alpha**	Pike silverside		
* A0A6J2PC09_COTGO	*Cottoperca gobio*	2 (2)	60
**nesprin-2**	Channel bull blenny		
* A0A0S7MGP3_9TELE	*Poeciliopsis prolifica*	3 (2)	59
**ZN287 (Fragment)**	Blackstripe livebearer		
* A0A3Q3VSX4_MOLML	*Mola mola*	1 (1)	59
**Uncharacterized protein**	Ocean sunfish		
* A0A553Q8B1_9TELE	*Danionella translucida*	3 (2)	58
**Uncharacterized protein**	Micro glassfish (Cyprinid)		
* A0A0P7TM62_SCLFO	*Scleropages formosus*	1 (1)	58
**Keratin, type I cytoskeletal 18-like**	Asian arowana		
* A0A060XKV1_ONCMY	*Oncorhynchus mykiss*	3 (2)	58
**[Histone H3]-trimethyl-L-lysine(9) demethylase**	Rainbow trout		
* E7F6Y7_DANRE	*Danio rerio*	4 (2)	58
**DNA polymerase kappa**	Zebrafish		
* F8W5U5_DANRE	*Danio rerio*	2 (2)	58
**Centrosomal protein of 290 kDa**	Zebrafish		
* A0A2U9CTT6_SCOMX	*Scophthalmus maximus*	7 (2)	57
**Putative utrophin**	Turbot		
* A0A3B3BVC4_ORYME	*Oryzias melastigma*	2 (2)	57
**Uncharacterized protein**	Marine medaka		
* A0A3B4UZF1_SERDU	*Seriola dumerili*	1 (1)	57
**[Histone H3]-lysine(4) N-trimethyltransferase **	Greater amberjack		
A0A060VW86_ONCMY	*Oncorhynchus mykiss*	1 (1)	56
**Uncharacterized protein (Tubulin alpha, tubulin domain containing)**	Rainbow trout		
* A0A671TLU7_SPAAU	*Sparus aurata*	3 (2)	56
**Reverse transcriptase**	Gilt-head bream		
A0A3Q4H8B0_NEOBR	*Neolamprologus brichardi*	1 (1)	56
**Ig-like domain-containing protein**	Lyretail cichlid		
* A0A0U2ERZ3_CORCL	*Coregonus clupeaformis*	6 (1)	56
**Glyceraldehyde 3-phosphate dehydrogenase**	Lake whitefish		
* A0A0R4IVM1_DANRE	*Danio rerio*	11 (2)	55
**LSM14A mRNA-processing body assembly factor b**	Zebrafish		
* A0A3P8VC95_CYNSE	*Cynoglossus semilaevis*	1 (1)	54
**Uncharacterized protein**	Tongue sole		
* Q9DFN6_GILMI	*Gillichthys mirabilis*	1 (1)	54
**Glyceraldehyde-3-phosphate dehydrogenase**			
* A0A3B3BWJ2_ORYME	*Oryzias melastigma*	2 (2)	54
**Uncharacterized protein**	Marine medaka		
* A0A6A4SGZ4_SCOMX	*Scophthalmus maximus*	1 (1)	54
**C1q domain-containing protein**	Turbot		
* A0A3B4EJ56_PYGNA	*Pygocentrus nattereri*	2 (2)	54
**von Willebrand factor**	Red-bellied piranha		
* A0A1S3RE28_SALSA	*Salmo salar*	1 (1)	53
**uncharacterized protein LOC106602330 isoform X1**	Atlantic salmon		
* A0A2I4CMN8_9TELE	*Austrofundulus limnaeus*	2 (2)	53
**titin-like**	Killifish		

^ⱡ^ Ions score is −10*Log(P), where P is the probability that the observed match is a random event. Individual ions scores > 53 indicate identity or extensive homology (*p* < 0.05). Protein scores are derived from ions scores as a non-probabilistic basis for ranking protein hits.

## Data Availability

Data is contained within the article and supplementary material.

## Supplementary Materials

The following are available online at https://www.mdpi.com/1422-0067/22/2/875/s1, Table S1: Deiminated proteins in serum-EVs of halibut (*Hippoglossus hippoglossus* L.), as identified by F95-enrichment in conjunction with LC-MS/MS analysis; full LC-MS/MS data. Table S2: Total proteins in serum-EVs of halibut (*Hippoglossus hippoglossus* L.); full LC-MS/MS data.
